# Theory driven analysis of social class and health outcomes using UK nationally representative longitudinal data

**DOI:** 10.1186/s12939-020-01302-4

**Published:** 2020-10-28

**Authors:** Welcome Wami, Gerry McCartney, Mel Bartley, Duncan Buchanan, Ruth Dundas, Srinivasa Vittal Katikireddi, Rich Mitchell, David Walsh

**Affiliations:** 1 MRC/CSO Social and Public Health Sciences Unit, University of Glasgow, 200 Renfield Street, Glasgow, G2 3AX UK; 2 Present Address: Amsterdam Institute for Global Health and Development, Paasheuvelweg 25, 1105 BP Amsterdam, Netherlands; 3 Public Health Scotland, Meridian Court, 5 Cadogan Street, Glasgow, G2 6QE UK; 4 Institute of Epidemiology & Health, University College London, 1-19 Torrington Place, London, WC1E 7HB UK; 5 Public Health Scotland, Gyle Square, 1 South Gyle Crescent, Edinburgh, EH12 9EB UK; 6 Glasgow Centre for Population Health, Olympia Building, Bridgeton Cross, Glasgow, G40 2QH UK

**Keywords:** Health inequalities, Self-rated health, SF-36, Social class theory, Socio-economic position

## Abstract

**Background:**

Social class is frequently used as a means of ranking the population to expose inequalities in health, but less often as a means of understanding the social processes of causation. We explored how effectively different social class mechanisms could be measured by longitudinal cohort data and whether those measures were able to explain health outcomes.

**Methods:**

Using a theoretically informed approach, we sought to map variables within the National Child Development Study (NCDS) to five different social class mechanisms: social background and early life circumstances; habitus and distinction; exploitation and domination; location within market relations; and power relations. Associations between the SF-36 physical, emotional and general health outcomes at age 50 years and the social class measures within NCDS were then assessed through separate multiple linear regression models. R^2^ values were used to quantify the proportion of variance in outcomes explained by the independent variables.

**Results:**

We were able to map the NCDS variables to the each of the social class mechanisms except ‘Power relations’. However, the success of the mapping varied across mechanisms. Furthermore, although relevant associations between exposures and outcomes were observed, the mapped NCDS variables explained little of the variation in health outcomes: for example, for physical functioning, the R^2^ values ranged from 0.04 to 0.10 across the four mechanisms we could map.

**Conclusions:**

This study has demonstrated both the potential and the limitations of available cohort studies in measuring aspects of social class theory. The relatively small amount of variation explained in the outcome variables in this study suggests that these are imperfect measures of the different social class mechanisms. However, the study lays an important foundation for further research to understand the complex interactions, at various life stages, between different aspects of social class and subsequent health outcomes.

**Supplementary information:**

**Supplementary information** accompanies this paper at 10.1186/s12939-020-01302-4.

## Background

There is an extensive literature on social class and its associations with health inequality. However, often this is based on simply *ranking* populations (for example, by occupation-derived measures of social position) to describe differences in health. Therefore there is a clear, requirement to fully understand the complex social and economic relationships between different population groups, and how those manifest themselves in inequalities in different health outcomes [26, 33]. This is particularly important in order that the causal processes generating and perpetuating health inequalities are understood, and, so that corrective policies can be introduced [29]. Some studies have previously considered the empirical relationship between measures of social class and health, going beyond the use of social position measures to simply expose inequalities. For example, measures of working class power were found to be a better explanation of lower infant mortality, low birthweight and nonintentional injury rates compared to social capital measures [28]. Using Scottish data, it was found that occupational social class explained more of the variation in mortality outcomes than educational attainment, which the authors described as proxies for material and cultural mechanisms respectively [15]; and a study comparing different social position measures and mechanisms using English data found independent, strong, relationships across material, occupation and cultural mechanisms with a wide range of health risks such as blood pressure and obesity [3]. Finally, some other contributions have sought to place social class relations in the context of political economy more broadly [36].

Building on the work of Wright [42, 43], a recent paper proposed a new integrated model of such a ‘theorisation of class relations’ [26]. This proposes interacting and interdependent social class mechanisms, which represent different ways in which the class structure of societies generates differential experiences and impacts. Note that within this model social closure and opportunity hoarding, as well as the social background and early life circumstances of social groups, lead to the relative position of social groups. The five types of social class mechanism proposed within the paper can be summarised as follows:

1. *Social background and early life circumstances:* this is the intergenerational exposure to social class mechanisms and the differential opportunities this confers from birth (or even before), relating to exposures and position of people’s ancestors. It also represents the potential ‘critical period’ exposures to impacts on health after substantial lag times. As such, this mechanism encompasses the impacts of the other mechanisms detailed below in terms of the exposures individuals and groups experience when they are young.
2. *Habitus and distinction:* this theory was first described by Bourdieu [7], and is defined as the ways in which different social classes display cultural markers which differentiate each from one another. These markers are usually formed in childhood and often outlive changes in economic circumstances. Examples include accents, ways of dressing and knowledge of cultural references (sport, theatre, television, history, etc.).
3. *Exploitation and domination:* the processes through which some social classes control the lives and activities of other classes (domination) and acquire economic benefits from the labour of others (exploitation), as first articulated by Marx [25, 43]. This social class process is therefore focused on the waged economy and how class groups are treated differently within that setting. Social groups find themselves in this position due to the power relations and legal rules to which they are exposed (e.g. the relative strength of trade unions within a workplace, and trade union legislation more generally, can have a substantial influence on the extent to which exploitation and domination occur), because of the processes of social closure and opportunity hoarding, and due to their social background and early life circumstances.
4. *Location within market relations:* describes how some social groups can maintain their advantageous economic position over others, primarily through their position within the labour market and the pay differentials this confers. This means that they have greater financial resources to use for consumption and to obtain revenue flows through interest, dividends, economic rents and profits (e.g. through savings, share ownership and housing rental). As with ‘Exploitation and domination’ above, social groups find themselves in this position due to the processes of social closure and opportunity hoarding, as well as their social background and early life circumstances [39].
5. *Power relations:* describes the ability of members of different social groups to control their own affairs and those of others – thereby incorporating all of the other social processes described above [20, 24]. This includes the different sources of power, the form the power takes (including the social relations involved), the positions of power and the social relationships this confers, and the social spaces in which these power relations occur ([27] et al., forthcoming).

**Fig. 1:**
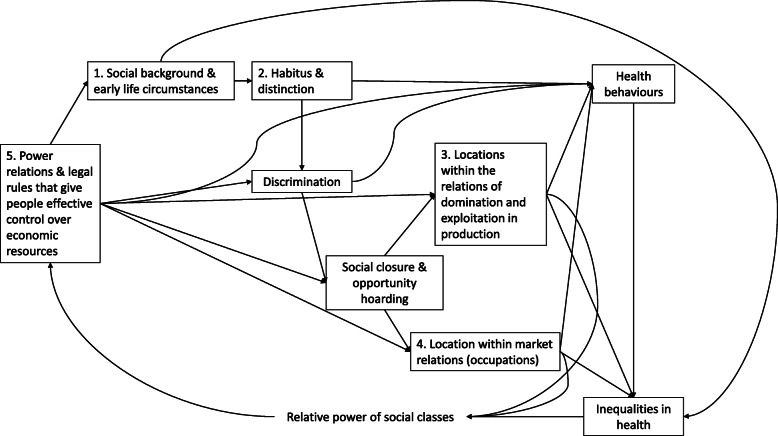
A representation of class relations to explore the different class mechanisms which explain inequalities in health outcomes. *Figure adapted from:* Mccartney, G., Bartley, M., Dundas, R., Katikireddi, S. V., Mitchell, R., Popham, F., Walsh, D. & Wami, W [25]. Theorising social class and its application to the study of health inequalities. *SSM - Population Health,* 10.1016/j.ssmph.2018.10.015

The connections between these different aspects of social class are shown in Fig. 1.

As with any such model, there is a need to test the extent to which the theory is supported by empirical data. The overarching aim of this study was to assess the extent to which this could be done using an appropriate longitudinal data set. Having longitudinal data is important because the various mechanisms often involve processes such as early life social advantage and acquisition of habitus that take place over the life course.

Specifically, the research questions were:

1. How effectively can longitudinal cohort data be mapped to the different social class mechanisms included in the model?
2. To what extent are the measurable aspects of the different social class mechanisms able to explain differences in a range of health outcomes? And to what extent does their explanatory power differ in relation to different outcomes?

## Methods

### Study population and selection of variables

The selected data set was the National Child Development Study (NCDS), also known as the 1958 British birth cohort study [31]. It was chosen as it is broadly representative of the wider British population, contains a broad range of relevant data, and has accrued many years of follow-up. The NCDS started in 1958 with a baseline cohort of more than 17,000 babies born in Scotland, England and Wales in one week of March 1958 [31]. The initial survey at birth has been followed by ten repeated follow-ups in 1965, 1969, 1974, 1981, 1991, 2000, 2003, 2004, 2008 and 2013 aimed at monitoring the health, development and social circumstances of the cohort members^1^ [12]. The study has collected detailed information on child, adolescent and adult social, economic and health-related circumstances over 50 years.

Building on the previous theoretical work regarding the links between different social class processes [26], we undertook an empirical analytical approach using indicators from the NCDS to test the potential utility of these theoretical class mechanisms in explaining health outcomes across populations. We took a pragmatic approach to provide a parsimonious best-fit of the available measures to the mechanisms presented in Fig. 1. As a first step, the NCDS’ data dictionaries were systematically examined to identify all potentially relevant measures of class. The quality and completeness of each chosen variable was then assessed, with some variables combined across different waves to create a single measure, as shown in Table 1. The sex variable (male/female) was included under each of the different aspects of social class theory. The data for all NCDS waves used in this study were sourced from the UK Data Service [12].

**Table 1:** Proposed measures of social class theory within the National Child Development Study (NCDS) cohort^a^

Social class mechanism	Proposed ‘best-fit’ measures within the NCDS longitudinal cohort study
**1. Social background and early life circumstances**	*•* ***Father’s Social Class:*** I; II; III-non-manual; III-manual; IV; V/other. Measured according to the Registrar-General’s schema which is regarded as an indicator of the prestige and skill levels of each occupation, at birth-1958 (Perinatal Mortality Survey). *•* ***Mother’s father (Grandparent’s) Social Class:*** I; II, III-non-manual; III-manual; IV; V/other. Measured at birth-1958 (Perinatal Mortality Survey). *•* ***Parental education:*** Was (a) mother, (b) father at school after minimum school-leaving age? Did not stay-16 years; did stay-16 years; did stay-16 to 18 years; did stay-19 or more years. Measured at birth-Perinatal Mortality Survey (PSM 1958). *•* ***Receipt of free school meals:*** Does any child in household/family get free school meals at any point during schooling? Yes/No. Derived from variables measured at childhood sweeps NCDS2 (1969) and NCDS3 (1974). *•* ***Family Financial hardships:*** number of times household experienced serious family financial hardships during cohort member’s childhood: 0 = none; 1 = at least once; 2 = at least twice. Derived from variables measured at childhood sweeps: NCDS1 (1965), NCDS2 (1969) and NCDS3 (1974). *•* ***Overcrowding (****defined as households with over 1.5 persons per room (excluding bathroom and kitchen*)): number of times cohort member experienced overcrowding in childhood: 0 = never; 1 = once; 2 = twice; 3 = at least 3 times. Derived from housing variables measured during childhood sweeps: NCDS1 (1965), NCDS2 (1969) and NCDS3 (1974). *•* ***Amenities:*** access to household amenities; indoor toilet, bathroom, kitchen: 0 = shared or no sole use; 1 = sole use of 1; 2 = sole use of 2; 3 = sole use of 3. Measured at survey sweep NCDS3 (1974).
**2. Habitus and distinction**	*•* ***Cognitive Ability measure*:** Draw-A-Man test score at age 7, as an indication of child’s general mental & perceptual ability (continuous variable). Measured at survey sweep NCDS1 (1965). *•* ***Teachers’ assessment of child’s ability*** *(derived ratings in Maths & English combined):* 1 = A-level & higher; 0 = other (below average CSE grades 2–4/ little ability O-level or CSE 1). Measured at survey sweep NCDS3 (1974). *•* ***Teacher’s view*:** ***child’s poor speech rating:*** 1 = not at all; 2 = somewhat; 3 = certainly; 4 = don’t know. Measured at survey sweep NCDS3 (1974). *•* ***Imagined occupation at age 25:*** 1 = higher managerial & professional; 2 = lower managerial & professional; 3 = intermediate occupations; 4 = small employers & own account workers; 5 = lower supervisory & technical; 6 = semi-routine occupations; 7 = routine; 8 = students, occupations not stated or inadequately described or not classified. Measured at survey sweep NCDS2 (1969). *•* ***Aspirations/plans after secondary school:*** 1 = full time studies; 2 = job, nothing more; 3 = part study & job; 4 = don’t know. Measured at survey sweep NCDS3 (1974). *•* ***Aspirations, where child wants to continue full-time study:*** 1 = university, polytechnic; 2 = college education; 3 = art college etc.; 4 = other college; 5 = somewhere else; 6 = don’t know. Measured at survey sweep NCDS3 (1974). *•* ***Leisure time activities:*** derived as a combined total number of leisure activities in childhood (0; 1; 2; 3; 4; 5 or more). Measured at survey sweep NCDS3 (1974). *•* ***TV watching:*** *Watched TV in the last 4 weeks?* 1 = 5 times a week+; 2 = 3–4 times a week; 3 = 1–2 times a week; 4 = 2–3 times last 4 weeks; 5 = Once in last 4 weeks; 5 = Not done in last 4 weeks. Measured at survey sweep NCDS3 (1974). *•* ***Book readership:*** *Read books in the past 4 weeks?* 1 = 5 times a week+; 2 = 3–4 times a week; 3 = 1–2 times a week; 4 = 2–3 times last 4 weeks; 5 = Once in last 4 weeks; 6 = Not done in last 4 weeks. Measured at survey sweep NCDS3 (1974). *•* ***Newspaper readership*:** 0 = none; 1 = Broadsheet (Telegraph, Financial Times, Guardian, Times); 2 = Tabloid (Daily papers- Express, Mail, Star, Evening, Mirror, Sun); 3 = other (regional etc.). Measured at survey sweep NCDS4 (1981). *•* ***Trades union membership/activity:*** Yes/No. Measured at survey sweep NCDS4 (1981). *•* ***Voting behaviour*** (party voted for in general elections)*:* 0 = none; 1 = Conservative; 2 = Labour; 3 = Liberal; 4 = Other (Welsh National, Scottish National, National Front, Communist, Workers Revolutionary Party (WRP)). Measured at survey sweep NCDS4 (1981). *•* ***Voting participation*** (current voting intentions): 0 = none/spoil; 1 = Conservative; 2 = Labour; 3 = Liberal; 4 = Social Democratic Party; 5 = other; 6 = Refused/Don’t Know. Measured at survey sweep NCDS4 (1981). *•* ***Religion:*** attendance at religious meetings: 0 = no religion; 1 = weekly or more; 2 = monthly or more; 3 = less than monthly; 4 = rarely or never. Measured at survey sweep NCDS4 (1981).
**3. Exploitation and domination**	**Note*:*** *most information on sources of own income including rental income, shares, investments (including amounts)* etc. *was captured at sweep NCDS4 (1981). Where available, we further cross-checked the reported information for consistency with data collected from other adulthood sweeps.* *•* ***Main finance used to buy home:*** 0 = none; 1 = Building Society; 2 = Mortgage/Loan; 3 = Other way (e.g. Sale property, Savings). Measured at survey sweep NCDS4 (1981). *•* ***Type of financial source of home purchase:*** 0 = none; 1 = Building Society Mortgage; 2 = Bank Mortgage/Loan; 3 = Other Loan (Local Authority Mortgage/Loan, Insurance Company Mortgage/Loan, Financial Company Mortgage/Loan, Loan-parents/in-laws, Government Home Loan Scheme, Other private loan); 4 = Gift/Inheritance; 5 = Other-way (Savings, sale of previous property). Measured at survey sweep NCDS4 (1981). *•* ***Mortgage as a percentage of house price:*** 1: < 50%; 2: 50–60%; 3: 60–70%; 4: 70–80%; 5: 80–90%; 6: 90–100%; 7: 100%. Measured at survey sweep NCDS4 (1981). *•* ***Housing tenure:*** 1 = own-outright; 2 = own-buying with help of a mortgage/loan; 3 = pay part rent and part mortgage (shared/equity ownership); 4 = rent it; 5 = live rent-free, including in relative’s / friend’s property; 6 = other; 7 = squatting. Measured at sweep NCDS8 (2008). Note that since this variable was captured throughout adulthood sweeps, we further checked survey sweeps NCDS4 (1981) - NCDS7 (2004) for completeness and consistency. *•* ***Dwelling size:*** number of rooms (apart from bathroom & kitchen)*:* 0; 1; 2; 3; 4; 5; 6 or more. Measured at survey sweep NCDS5 (1991). In the case of cohort members moving house, we cross-checked this information for consistency from other adulthood sweeps. *•* ***Value of inheritance or gift or loan:*** in pounds (£). Measured at survey sweep NCDS4 (1981). *•* ***Investments, savings and debt:*** in pounds (£). Measured at survey sweep NCDS4 (1981). *•* ***Capital accrued:*** derived from the difference between property purchase price and size of loan or gift and ranked: 1-least to 5-most capital accrued. Measured at survey sweep NCDS4 (1981).
**4. Location within market relations**	*•* ***Highest educational qualifications attained:*** 0 = none; 1 = NVQ1 level/low-grade GCSE/O-levels or equivalent; 2 = O-level A-C grade/NVQ3 level or equivalent; 3 = A-levels/NQV3 level or equivalent; 4 = Degree/NVQ4 level or equivalent; 5 = Higher degree/NVQ5 level or equivalent. Measured in survey sweep NCDS7 (2004). *•* ***Education attainment:*** age when cohort member left education. Measured at survey sweep NCDS6 (2000). *•* ***Type of Secondary School:*** 1 = Comprehensive; 2 = Grammar; 3 = Private (includes Independent, Direct grant, special schools); 4 = Secondary modern; 5 = Other (Unknown reason). Measured at survey sweep NCDS3 (1974). *•* ***Receipt of benefits:*** number of times cohort member received means-tested (e.g. unemployment, supplementary, family income support, family credit) benefits in adulthood*:* 0 = none; 1; 2; 3; 4 or more times. Derived from variables measured during adulthood sweeps: NCDS4 (1981) - NCDS8 (2008). *•* ***Income and partner’s income:*** Family net income per week (£). Measured at survey sweep NCDS5 (1991). *•* ***Debt and savings (own and partner’s):*** in pounds (£). Measured at survey sweep NCDS5 (1991). *•* ***Unemployment periods:*** cohort member’s number of episodes of unemployment in adulthood 0 = none; 1; 2; 3 or more. Derived from variables measured at adulthood sweeps with the record of type of benefits received identified: NCDS4 (1981) - NCDS8 (2008). *•* ***Own social class:*** Cohort member’s own social class. Measured according to the Registrar-General’s schema at sweep NCDS6 (2000).
**5. Power relations**	*No measures identified for this class theory.*

*Data Source:* University of London. UCL Institute of Education. Centre for Longitudinal Studies. National Child Development Study: 1958-. Colchester, Essex: UK Data Archive [distributor], September 2018. SN: 2000032. Retrieved from: https://discover.ukdataservice.ac.uk/series/?sn=2000032

^a^Sex variable (male/female) included in the analysis of each of the social class mechanisms

### Health outcome variables

The main outcome variables in our study were based on the Short Form (SF-36) Physical functioning, Emotional well-being, and General health domains measured at age 50, analysed separately as continuous variables for each social class theory. Briefly, SF-36 is a multi-purpose health survey instrument comprised of 36 questions, yielding a profile of functional health and well-being scores [32]. Each of the scales are scored between 0 and 100, with higher scores indicating better health [32, 38]. The three SF-36 domain scores selected as outcomes for this study were described within the NCDS as follows: *Physical functioning score:*- the lowest possible score indicated very limited physical activities, and the highest possible score indicated the individual was able to perform all types of physical activities without any limitations due to health; *Emotional well-being score*:- the lowest score indicated feelings such as nervousness and depression most of the time, and the highest score indicated good feelings such as being happy, peaceful and calm all the time; *General health score:* the lowest score indicated poor health and personal belief it was likely to get worse and the highest score evaluated health as excellent [21].

### Analytic sample and handling of missing data

The analytic sample was composed of 8787 individuals: these are cohort members who had at least one valid response for any of the SF-36 health domains measured at age 50 (in 2008). The percentage of male and female survey participants was 48.1 and 51.9%, respectively. Of these, only a very small proportion had missing data for the outcome variables investigated in this study: 0.2% (*n* = 17) for Physical functioning, 0.3% (*n* = 26) for Emotional well-being, and none for the General health outcome. However, some data were missing (to varying degrees) in relation to some of the explanatory variables shown in Table 1. Missing data were handled with a combination of multiple imputation and weighting [35]. Inverse probability weights were used to weight the analysis sample to the baseline sample to correct for bias and unequal sampling fractions across the different cohort survey waves [34]. The weights were derived from a logistic regression of having valid outcome data on factors associated with drop-out (gender, head of household social class at birth, maternal smoking and parity). Missing data within the analytic sample were addressed using multiple imputation. We created 20 different datasets accounting for SF-36 health outcomes, sex, and the inverse probability weights in the imputation model.

### Statistical analysis

Descriptive summary statistics (sample means and standard deviations [SD]) were used to explore the distribution of the three SF-36 health outcomes. For each of the proposed social class mechanisms, a set of three separate multiple regression models were then fitted to assess the extent to which the exposure variables in Table 1 (including sex) explained subsequent health outcomes. Both complete case (results in Additional File 1) and multiple imputation analysis (results reported here) were conducted. The extent to which variation in the SF-36 health outcomes across the whole sample was explained by the different sets of predictor variables (i.e. for each social class theory) was assessed by means of the *R*-squared values (*R*^*2*^). As one of the aims of the study was to assess how effectively different NCDS variables could be mapped to the proposed social mechanisms, and was not seeking to find a best-fit statistical model, interactions between the explanatory variables were not considered. For each of the weighted regression models that were estimated with multiply imputed data, the combined R-squared values together with their corresponding 95% Confidence Intervals (95% CI) were determined using the Fisher’s r to z transformation technique [6, 19]. Adjusted predicted means (least-squares means) [95% CIs] and regression coefficients [SEs], were reported for all exposure variables. The validity of the SF-36 variables within this cohort was checked by means of comparison with the self-assessed general health variable and a range of disease-specific variables. The results are not reported within this paper but are available within the online appendix (see Additional File 2). All modelling was carried out in SAS version 9.4 (SAS Institute Inc., Cary, NC, USA).

## Results

### Mapping of variables to social class mechanisms

There are a number of exposure variables within the NCDS which are potentially relevant for exploring the class mechanisms above. However, as the data were obviously collected for purposes other than an analysis of social class processes, many aspects of class theory were not measured, and many of the available measures could legitimately be applied to multiple mechanisms. Obvious areas where there is a lack of appropriate variables include measures of social distinction (such as accent and the ability to ‘fit in’ to a wide range of social settings), good measures of the ownership of economic capital and the experience of discrimination. No NCDS variables were identified as relevant to the ‘*Power relations*’ theory. The suggested measures included in Table 1 are therefore a pragmatic application of the available data to the different social class mechanisms. We included measures of social closure and opportunity hoarding within theory 4 (location within market relations) rather than analysing separately (this particularly refers to education measures). Arguably these measures could be included in mechanisms 3 (exploitation and domination) and 4, or could have been analysed separately.

### Exploring SF-36 health outcome scores

The proportion of cohort members with highest achievable SF-36 score of 100 (ceiling effect) was 36% for Physical functioning, 3% for Emotional well-being and 7% for General health. The majority of these cohort respondents had high SF-36 scores (above 60 points) (data not shown). A small proportion (< 1%) reported extreme poor health i.e. a minimum possible score of zero in the SF-36 scale (floor effect). The overall sample mean SF-36 scores were as follows - Physical functioning: mean = 86.1 (SD = 21.7); Emotional well-being: mean = 75.0 (SD = 18.1); and General health: mean = 68.3 (SD = 22.0). Men tended to have slightly higher scores than women for physical functioning (difference in means = 3.3; 95% CI: 2.4–4.2) and Emotional well-being (difference in means = 3.0; 95% CI: 2.2–3.8), but not General health, scores for which were similar for men and women (difference in means = 1.0; 95% CI: 0.1–1.9). Comparisons of the three SF-36 outcomes with cohort members’ self-assessed health showed that those who rated themselves as having at least good health or much better health (compared to a year ago) had higher mean SF-36 scores compared to those who said their health was poor or much worse (Additional File 2, Figure S1).

### Assessing measurable aspects of different social class mechanisms within the NCDS

Tables 2, 3, 4 and 5 shows results of the fully-adjusted regression models for each social class theory, including the corresponding parameter estimates and adjusted mean SF-36 scores predictors included in the models. F-tests of overall significance in the regression analyses are shown in Table S2 (in Additional File 2). Overall, there was a relatively small amount of variation explained by the predictors in all the models, as measured by the *R*^*2*^ statistic. Among the different proposed measures of class theory, ‘*Social background and early life circumstances*’ explained the least variation in the three outcomes: at most only 4% of total variability in the outcome scores. ‘*Location within market relations*’ explained the most variation, although again the total amount of variability explained was low across all models: 10% for Physical functioning, 8% for General health and 7% for Emotional well-being. The following summarises the results for each theory/set of models:

1. ‘Social background and early life circumstances’ class theory:

**Table 2:** ‘*Social background and early life circumstances*’ class theory: associations between SF-36 health outcomes at age 50 and measures of class theory. Parameter estimates and predicted means (LSMeans) from multiply imputed and weighted regression modelling

	Physical functioning		Emotional well-being		General health	
Variable	Estimate (SE)	Mean (95% CI)	Estimate (SE)	Mean (95% CI)	Estimate (SE)	Mean (95% CI)
***Intercept***	82.4 (2.5)	–	67.8 (2.0)	–	61.4 (2.5)	–
***Gender***						
Female	−3.3 (0.5)	82 (80, 84)	−3.0 (0.4)	71 (69, 73)	1.1 (0.5)	66 (64, 68)
Male	(ref)	86 (83, 88)	(ref)	74 (72, 76)	(ref)	65 (63, 67)
***Father’s Social Class***						
Professional (I)	5.2 (1.5)	86 (83, 89)	2.4 (1.2)	73 (71, 75)	6.1 (1.5)	68 (65, 71)
Managerial-technical (II)	4.5 (1.2)	86 (83, 88)	2.1 (1.0)	73 (71, 75)	4.8 (1.2)	67 (65, 69)
Skilled non-manual (III-NM)	4.2 (1.2)	85 (83, 88)	2.7 (1.0)	73 (71, 76)	4.6 (1.2)	67 (64, 69)
Skilled-manual (III-M)	2.0 (1.0)	83 (81, 85)	1.4 (0.8)	72 (70, 74)	2.6 (1.0)	65 (63, 67)
Partly skilled (IV)	1.1 (1.1)	82 (80, 84)	1.8 (0.9)	72 (70, 74)	2.2 (1.0)	64 (62, 67)
Unskilled & Other (V/Other)	(ref)	81 (79, 83)	(ref)	71 (69, 73)	(ref)	62 (60, 65)
***Grandparent’s Social Class***						
Professional (I)	−0.4 (1.9)	84 (80, 87)	0.4 (1.7)	73 (69, 76)	−1.2 (1.9)	65 (61, 69)
Managerial-technical (II)	−0.5 (1.2)	84 (81, 86)	1.1 (0.9)	73 (71, 75)	−0.3 (1.2)	66 (64, 68)
Skilled non-manual (III-NM)	−0.4 (1.4)	84 (82, 87)	0.5 (1.2)	73 (70, 75)	−0.4 (1.5)	66 (63, 69)
Skilled-manual (III-M)	−0.5 (1.0)	84 (81, 86)	−0.2 (0.8)	72 (70, 74)	−1.6 (1.0)	65 (63, 67)
Partly skilled (IV)	−0.3 (1.0)	84 (81, 86)	−0.1 (0.8)	72 (70, 74)	−0.9 (1.0)	65 (63, 68)
Unskilled & Other (V/Other)	(ref)	84 (81, 87)	(ref)	72 (70, 74)	(ref)	66 (64, 69)
***Mother’s education (Did stay at school after min. Leaving age?)***						
Did not stay-16 years	−3.0 (1.6)	82 (80, 84)	−1.7 (1.4)	71 (70, 73)	−2.2 (1.7)	64 (62, 66)
Did stay-16 years	−1.7 (1.7)	84 (81, 86)	0.1 (1.4)	73 (71, 75)	−0.6 (1.7)	66 (63, 68)
Did stay-16 to 18 years	−1.0 (1.6)	84 (82, 87)	−1.1 (1.4)	72 (70, 74)	0.2 (1.7)	66 (64, 69)
Did stay-19 or more years	(ref)	85 (82, 89)	(ref)	73 (70, 76)	(ref)	66 (63, 70)
***Father’s education (Did stay at school after min. Leaving age?)***						
Did not stay-16 years	−2.3 (1.4)	82 (80, 85)	0.9 (1.2)	73 (71, 74)	−2.4 (1.5)	64 (62, 67)
Did stay-16 years	−1.7 (1.4)	83 (81, 85)	0.5 (1.3)	72 (70, 74)	−2.4 (1.5)	64 (62, 67)
Did stay-16 to 18 years	0.3 (1.5)	85 (83, 87)	1.3 (1.2)	73 (71, 75)	−0.6 (1.5)	66 (64, 69)
Did stay-19 or more years	(ref)	85 (82, 88)	(ref)	72 (69, 74)	(ref)	67 (64, 70)
***Receipt of free school meals by any child in the household***						
No	3.4 (0.8)	86 (83, 88)	1.9 (0.7)	73 (71, 75)	2.7 (0.8)	67 (65, 69)
Yes	(ref)	82 (80, 84)	(ref)	71 (70, 73)	(ref)	64 (62, 66)
***Experienced family financial hardships in childhood***						
Never	2.6 (1.4)	86 (84, 88)	4.6 (1.2)	75 (73, 76)	5.1 (1.4)	68 (66, 70)
Once	−0.3 (1.4)	83 (81, 85)	2.4 (1.2)	72 (71, 74)	1.8 (1.4)	65 (63, 67)
Twice or more	(ref)	83 (80, 86)	(ref)	70 (67, 73)	(ref)	63 (60, 66)
***Experienced overcrowding in childhood***						
Never	2.8 (1.2)	85 (83, 88)	1.8 (1.0)	73 (71, 75)	1.1 (1.2)	66 (64, 68)
Once	0.8 (1.3)	84 (81, 86)	1.2 (1.1)	72 (70, 74)	0.6 (1.3)	65 (63, 68)
Twice	1.0 (1.5)	84 (81, 86)	1.8 (1.2)	73 (71, 75)	0.9 (1.5)	66 (63, 68)
Three times or more	(ref)	83 (80, 86)	(ref)	71 (69, 74)	(ref)	65 (62, 68)
***Access to household amenities***^a^						
Shared or no sole use	1.1 (2.1)	86 (81, 90)	1.6 (1.8)	74 (71, 78)	1.4 (2.1)	68 (64, 73)
Sole use of 1	−2.7 (1.9)	82 (78, 86)	−2.5 (1.6)	70 (67, 74)	−4.6 (1.9)	62 (58, 66)
Sole use of 2	−1.4 (1.4)	83 (80, 86)	−0.8 (1.2)	72 (69, 75)	−2.3 (1.4)	65 (62, 68)
Sole use of 3	(ref)	85 (83, 86)	(ref)	73 (72, 74)	(ref)	67 (65, 68)

^a^Access to household amenities: indoor toilet, bathroom, kitchen

*SE* standard error, *ref* reference category

**Table 3:** ‘*Habitus and distinction*’ class theory: associations between SF-36 health outcomes at age 50 and measures of class theory. Parameter estimates and predicted means (LSMeans) from multiply imputed and weighted regression modelling

	Physical functioning		Emotional well-being		General health	
Variable	Estimate (SE)	Mean (95% CI)	Estimate (SE)	Mean (95% CI)	Estimate (SE)	Mean (95% CI)
***Intercept***	76.7 (4.2)	–	70.3 (3.4)	–	60.0 (4.0)	–
***Gender***						
Female	−4.4 (0.5)	80 (75, 85)	−3.2 (0.4)	71 (66, 76)	0.4 (0.5)	66 (61, 72)
Male	(ref)	85 (79, 90)	(ref)	74 (69, 79)	(ref)	66 (60, 71)
***Cognitive Ability (Draw-A-Man Test score)***	0.2 (0.04)	–	0.1 (0.03)	–	0.2 (0.04)	–
***Number of leisure time activities in childhood***						
0	−1.2 (3.8)	83 (74, 91)	−1.2 (2.4)	74 (67, 80)	−2.2 (3.9)	65 (55, 75)
1	−6.3 (4.9)	78 (67, 88)	−9.4 (6.0)	65 (53, 78)	−4.7 (4.9)	62 (52, 73)
2	−2.1 (1.8)	82 (76, 88)	−0.9 (1.6)	74 (69, 79)	1.7 (1.9)	69 (62, 75)
3	− 0.8 (1.1)	83 (78, 89)	− 0.9 (0.9)	74 (69, 78)	− 0.8 (1.1)	66 (61, 72)
4	−0.2 (0.7)	84 (79, 89)	−1.2 (0.6)	74 (69, 78)	− 0.1 (0.8)	67 (62, 72)
5 or more	(ref)	84 (79, 89)	(ref)	75 (71, 79)	(ref)	67 (62, 72)
***Imagined occupation at age 25***						
Higher managerial & professional	1.7 (1.2)	83 (77, 88)	−0.7 (1.0)	71 (66, 75)	−0.5 (1.2)	66 (60, 71)
Lower managerial & professional	2.2 (0.9)	83 (78, 88)	0.8 (0.8)	72 (67, 77)	1.3 (0.9)	67 (62, 73)
Intermediate occupations	1.5 (1.0)	83 (77, 88)	1.3 (0.9)	73 (68, 77)	0.7 (1.0)	67 (61, 72)
Small employers & Own account workers	−0.2 (4.0)	81 (72, 90)	2.9 (3.8)	74 (66, 83)	−2.2 (4.1)	64 (54, 74)
Lower supervisory & Technical	0.8 (1.0)	82 (77, 87)	2.2 (0.9)	74 (69, 78)	0.2 (1.1)	66 (61, 72)
Semi-routine occupations	0.6 (1.0)	82 (76, 87)	− 0.6 (0.9)	71 (66, 76)	−0.4 (1.1)	66 (60, 71)
Routine	3.5 (1.4)	85 (79, 90)	2.6 (1.1)	74 (69, 79)	1.1 (1.4)	67 (61, 73)
Students/Not stated/Inadequately described/Not classified	(ref)	81 (76, 86)	(ref)	71 (67, 76)	(ref)	66 (61, 72)
***Aspiration after secondary school***						
Don’t know	−1.8 (0.9)	82 (76, 87)	−0.8 (0.6)	72 (67, 77)	−0.9 (0.8)	66 (60, 71)
Full time studies	−0.7 (0.8)	83 (77, 88)	0.1 (0.6)	73 (68, 78)	0.4 (0.8)	67 (61, 73)
Job, nothing more	−2.0 (0.8)	81 (76, 87)	−1.0 (0.7)	72 (67, 77)	−1.6 (0.8)	65 (59, 71)
Part study, job	(ref)	83 (78, 89)	(ref)	73 (68, 78)	(ref)	67 (61, 72)
***Teachers’ rating: child’s poor speech***						
Certainly	−10.2 (4.7)	73 (64, 82)	−3.9 (3.8)	67 (60, 75)	−1.3 (4.4)	64 (55, 73)
Don’t know	5.8 (8.2)	89 (73, 100^a^)	6.4 (7.0)	78 (64, 92)	1.4 (8.6)	67 (50, 84)
Not at all	2.4 (1.1)	85 (82, 88)	2.2 (0.9)	73 (71, 76)	2.4 (1.1)	68 (65, 71)
Somewhat	(ref)	83 (79, 86)	(ref)	71 (68, 74)	(ref)	65 (62, 69)
***Teachers’ ability rating: Maths & English***						
Little ability/below average CSE grades 2–4/O-level/CSE 1	−3.9 (0.7)	80 (75, 86)	−0.1 (0.7)	72 (68, 77)	−2.5 (0.7)	65 (59, 70)
A-level and higher	(ref)	84 (79, 90)	(ref)	73 (68, 77)	(ref)	67 (62, 73)
***TV watching***						
1–2 times a week	7.1 (3.8)	84 (79, 89)	3.4 (2.4)	74 (69, 79)	7.8 (2.8)	68 (62, 73)
2–3 times in last 4 weeks	6.5 (3.3)	83 (78, 89)	2.9 (2.8)	73 (68, 78)	7.6 (3.5)	68 (62, 74)
3–4 times a week	7.2 (3.2)	84 (78, 89)	3.3 (2.5)	74 (69, 79)	7.7 (3.2)	68 (62, 73)
5 times a week +	5.4 (3.0)	82 (77, 87)	1.8 (2.4)	72 (68, 77)	5.4 (2.8)	66 (60, 71)
Not done in last 4 weeks	6.9 (3.1)	84 (77, 91)	0.8 (3.1)	71 (65, 77)	7.5 (3.0)	68 (61, 75)
One in last 4 weeks	(ref)	77 (69, 84)	(ref)	70 (64, 77)	(ref)	60 (52, 68)
***Book readership***						
1–2 times a week	−0.1 (1.0)	83 (77, 88)	0.3 (0.9)	72 (68, 77)	−1.5 (1.0)	66 (60, 72)
2–3 times in last 4 weeks	−0.6 (1.2)	82 (77, 88)	0.5 (1.0)	73 (68, 78)	−1.5 (1.0)	66 (60, 72)
3–4 times a week	−0.2 (1.1)	83 (77, 88)	0.6 (0.8)	73 (68, 78)	−1.6 (1.1)	66 (60, 71)
5 times a week +	−0.3 (1.1)	83 (78, 88)	0.5 (0.9)	73 (68, 77)	−1.7 (1.2)	66 (60, 72)
Not done in last 4 weeks	−2.9 (0.9)	80 (75, 85)	0.1 (0.7)	72 (68, 77)	−1.7 (0.9)	66 (60, 71)
One in last 4 weeks	(ref)	83 (78, 88)	(ref)	72 (67, 77)	(ref)	67 (62, 73)
***Newspaper readership***						
Tabloid (Daily papers-Express, Mail, Star, Evening, Mirror, Sun)	0.8 (0.9)	83 (78, 89)	1.0 (0.7)	73 (69, 78)	1.1 (0.9)	67 (62, 73)
Broadsheet (Telegraph, Fin. Times, Guardian, Times)	−0.9 (0.8)	82 (76, 87)	−0.6 (0.6)	72 (67, 77)	−1.4 (0.8)	65 (59, 70)
Other (e.g. Newsline, Regional etc.)	−1.2 (0.7)	81 (76, 87)	−0.2 (0.6)	72 (67, 77)	0.0 (0.7)	66 (60, 72)
None	(ref)	83 (77, 88)	(ref)	72 (68, 77)	(ref)	66 (61, 72)
***Voting behaviour (party voted for in last general elections)***						
Conservative	2.0 (1.8)	83 (78, 88)	−1.6 (1.5)	72 (67, 77)	0.3 (1.9)	67 (61, 72)
Labour	0.5 (1.8)	82 (76, 87)	−1.8 (1.5)	72 (67, 77)	−1.1 (1.9)	65 (60, 71)
Liberal	2.1 (1.9)	83 (78, 89)	−1.1 (1.6)	73 (68, 78)	−0.2 (1.9)	66 (61, 72)
Other (Welsh Nat, Scots Nat, Nat front, Communist, WRP)	1.1 (1.7)	82 (77, 88)	−2.6 (1.5)	71 (67, 76)	−0.8 (1.8)	66 (60, 71)
None	(ref)	81 (75, 87)	(ref)	74 (69, 79)	(ref)	66 (60, 73)
***Current voting intentions***						
Conservative	2.3 (0.9)	84 (79, 90)	2.0 (0.7)	75 (70, 79)	2.2 (0.9)	68 (63, 74)
Labour	−0.1 (0.8)	82 (77, 87)	−0.4 (0.6)	72 (67, 77)	−0.5 (0.8)	66 (60, 71)
Liberal	1.3 (1.1)	83 (78, 89)	−0.5 (0.9)	72 (67, 77)	0.5 (1.1)	67 (61, 73)
Social DP	−1.3 (0.9)	81 (75, 86)	0.1 (0.8)	73 (68, 78)	−1.0 (1.0)	65 (60, 71)
Other	−2.3 (1.7)	80 (74, 86)	−2.5 (1.4)	70 (65, 76)	−3.6 (1.7)	63 (56, 69)
None/Spoil	2.2 (1.1)	84 (79, 90)	−0.4 (0.9)	72 (67, 77)	1.6 (1.1)	68 (62, 74)
Refused/Don’t Know	(ref)	82 (77, 87)	(ref)	73 (68, 78)	(ref)	66 (61, 72)
***Trades union membership***						
Yes	−0.1 (0.5)	82 (77, 88)	−0.1 (0.4)	72 (68, 77)	−0.4 (0.5)	66 (60, 71)
No	(ref)	82 (77, 87)	(ref)	73 (68, 77)	(ref)	66 (61, 72)
***Religion (attendance at religious meetings)***						
Less than monthly	−0.8 (1.3)	82 (76, 87)	1.3 (1.0)	73 (68, 78)	−0.2 (1.2)	66 (60, 72)
Monthly or More	1.3 (1.7)	84 (78, 89)	1.1 (1.4)	73 (68, 78)	1.9 (1.7)	68 (62, 74)
No religion	−0.6 (1.0)	82 (76, 87)	0.2 (0.9)	72 (67, 77)	−0.9 (1.0)	65 (60, 71)
Rarely or Never	0.2 (1.0)	83 (77, 88)	0.2 (0.8)	72 (67, 77)	−0.7 (1.0)	65 (60, 71)
Weekly or More	(ref)	82 (77, 88)	(ref)	72 (67, 77)	(ref)	66 (60, 72)

^a^Rounded-off to the maximum achievable score

*Nat* National, *WRP* Workers Revolutionary Party, *Social DP* Social Democratic Party

**Table 4:** ‘*Exploitation and domination*’ class theory: associations between SF-36 health outcomes at age 50 and measures of class theory. Parameter estimates and predicted means (LSMeans) from multiply imputed and weighted regression modelling

	Physical functioning		Emotional well-being		General health	
Variable	Estimate (SE)	Mean (95% CI)	Estimate (SE)	Mean (95% CI)	Estimate (SE)	Mean (95% CI)
***Intercept***	100.0^a^ (21.0)	–	83.1 (17.6)	–	51.4 (21.2)	–
***Gender***						
Female	−3.5 (0.5)	83 (73, 94)	−3.2 (0.4)	71 (63, 80)	0.8 (0.5)	64 (54, 74)
Male	(ref)	87 (76, 97)	(ref)	75 (66, 83)	(ref)	63 (53, 73)
***Capital accrued (ranks)***						
1 (least)	−3.6 (2.7)	83 (72, 94)	−1.8 (1.8)	72 (63, 81)	−4.3 (2.5)	62 (51, 72)
2	−3.4 (2.3)	84 (73, 94)	−2.3 (1.7)	71 (63, 80)	−4.3 (2.1)	62 (51, 72)
3	−1.4 (2.0)	86 (75, 96)	−0.9 (1.6)	73 (64, 82)	−2.2 (1.9)	64 (53, 74)
4	−0.9 (1.8)	86 (75, 97)	1.0 (1.4)	75 (66, 83)	−0.8 (1.8)	65 (55, 75)
5 (most)	(ref)	87 (76, 97)	(ref)	74 (66, 82)	(ref)	66 (56, 76)
***Main finance used to by home***						
None	0.0 (2.1)	85 (75, 95)	−0.5 (1.9)	73 (64, 81)	0.5 (2.1)	63 (53, 74)
Building Society	0.4 (2.0)	85 (75, 96)	0.3 (1.7)	73 (65, 82)	1.9 (1.9)	65 (55, 75)
Mortgage/Loan	0.3 (2.2)	85 (74, 96)	−0.2 (1.9)	73 (64, 82)	0.5 (2.0)	63 (53, 74)
Other ways (e.g. Sale property, Savings)	(ref)	85 (75, 95)	(ref)	73 (64, 82)	(ref)	63 (53, 73)
***Type of financial source for home purchase***						
None	1.5 (24.6)	85 (36, 100^a^)	3.4 (18.9)	76 (38, 113)	11.1 (22.9)	72 (27, 118)
Building Society Mortgage	1.7 (2.6)	85 (79, 92)	0.6 (1.2)	73 (67, 78)	1.1 (2.2)	62 (56, 69)
Bank Mortgage/Loan	2.3 (3.1)	86 (79, 93)	0.5 (1.3)	73 (67, 78)	1.8 (2.7)	63 (56, 70)
Other Loan (e.g. Private, Government Homeloan)	1.4 (2.7)	85 (78, 92)	0.5 (1.4)	73 (67, 78)	0.9 (2.1)	62 (55, 69)
Gift/Inheritance	1.7 (4.1)	85 (77, 94)	0.0 (2.4)	72 (65, 79)	−0.2 (3.4)	61 (53, 69)
Other ways (e.g. Sale of previous property, Savings)	(ref)	84 (76, 91)	(ref)	72 (67, 78)	(ref)	61 (54, 68)
***Household size (excl. Bathroom & kitchen)***						
0	−4.8 (2.5)	83 (71, 95)	−4.8 (2.2)	71 (62, 81)	−1.8 (2.6)	65 (53, 76)
2	−5.7 (3.5)	82 (70, 94)	−5.8 (2.9)	70 (60, 80)	−9.6 (3.5)	57 (45, 69)
3	− 1.9 (1.6)	86 (75, 97)	−3.8 (1.3)	73 (64, 81)	−1.5 (1.6)	65 (55, 75)
3	−2.9 (1.0)	85 (75, 95)	−3.9 (0.7)	72 (64, 81)	−3.0 (0.9)	63 (54, 73)
4	−2.3 (0.7)	86 (75, 96)	−2.5 (0.6)	74 (65, 82)	−2.4 (0.7)	64 (54, 74)
5	−2.0 (0.7)	86 (76, 96)	−1.8 (0.6)	74 (66, 83)	−2.2 (0.7)	64 (54, 74)
6	(ref)	88 (77, 98)	(ref)	76 (68, 85)	(ref)	66 (57, 76)
***Mortgage as a percent of house price***						
100%	1.3 (2.5)	86 (75, 97)	0.3 (2.0)	73 (65, 82)	2.9 (3.7)	65 (54, 75)
90–100%	3.3 (2.8)	88 (77, 98)	1.3 (2.2)	74 (66, 82)	4.3 (2.8)	66 (56, 76)
80–90%	0.8 (2.7)	85 (75, 95)	0.8 (2.4)	73 (65, 81)	0.7 (2.7)	63 (53, 73)
70–80%	1.7 (3.3)	86 (75, 96)	−0.2 (2.0)	72 (63, 80)	0.8 (2.5)	63 (53, 73)
60–70%	0.6 (2.8)	85 (74, 96)	−1.0 (1.9)	72 (63, 81)	1.8 (2.5)	64 (53, 74)
50–60%	−1.9 (3.2)	82 (71, 94)	1.7 (2.2)	74 (64, 83)	1.1 (2.4)	63 (51, 74)
Under 50%	(ref)	84 (72, 96)	(ref)	73 (64, 82)	(ref)	62 (51, 73)
***Housing tenure***						
Live rent-free, incl. in relatives/friends property	−17.3 (20.9)	80 (71, 90)	−6.8 (17.5)	72 (64, 79)	13.1 (21.2)	64 (56, 73)
Other	−9.3 (21.0)	88 (78, 99)	−5.4 (17.6)	73 (65, 81)	18.7 (21.3)	70 (60, 80)
Own - buying with help of mortgage/loan	−11.1 (20.8)	87 (78, 95)	−3.1 (17.5)	76 (69, 83)	19.3 (21.1)	71 (63, 79)
Own - outright	−10.9 (20.8)	87 (78, 95)	−2.7 (17.5)	76 (69, 82)	18.1 (21.1)	69 (61, 77)
Pay part rent/part mortgage (shared/equivalent of)	−17.3 (21.1)	80 (69, 91)	−9.5 (17.7)	69 (60, 78)	9.7 (21.3)	61 (50, 72)
Rent it	−22.8 (20.9)	75 (66, 84)	−11.6 (17.5)	67 (60, 74)	6.6 (21.1)	58 (50, 66)
Squatting	(ref)	98 (56, 100^a^)	(ref)	79 (44, 113)	(ref)	51 (9, 93)

^a^Rounded-off to the maximum achievable score

**Table 5:** ‘*Location within market relations*’ class theory: associations between SF-36 health outcomes at age 50 and measures of class theory. Parameter estimates and predicted means (LSMeans) from multiply imputed and weighted regression modelling

	Physical functioning		Emotional well-being		General health	
	Estimate (SE)	Mean (95% CI)	Estimate (SE)	Mean (95% CI)	Estimate (SE)	Mean (95% CI)
***Intercept***	61.0 (4.0)		60.4 (3.4)		38.3 (4.1)	
***Gender***						
Female	−2.6 (0.5)	76 (75, 78)	−2.2 (0.4)	68 (66, 69)	1.9 (0.5)	60 (59, 62)
Male	(ref)	79 (78, 80)	(ref)	70 (69, 71)	(ref)	58 (57, 60)
***Own Social Class***						
Professional (I)	6.0 (3.0)	82 (80, 84)	5.1 (2.3)	71 (70, 73)	7.0 (2.7)	63 (61, 65)
Managerial-technical (II)	4.3 (2.9)	80 (79, 82)	5.2 (2.2)	71 (70, 72)	6.1 (2.7)	62 (61, 64)
Skilled non-manual (III-NM)	2.7 (2.9)	79 (77, 80)	3.4 (2.2)	69 (68, 71)	4.4 (2.7)	61 (59, 62)
Skilled-manual (III-M)	1.1 (2.9)	77 (76, 79)	4.6 (2.2)	71 (69, 72)	4.2 (2.7)	60 (59, 62)
Partly skilled (IV)	−4.3 (2.9)	72 (69, 74)	−2.4 (2.4)	64 (61, 66)	−2.8 (2.9)	53 (51, 56)
Unskilled & Other (V/Other)	(ref)	76 (71, 82)	(ref)	66 (62, 70)	(ref)	56 (51, 61)
***Type of Secondary School***						
Comprehensive	2.4 (1.7)	78 (76, 79)	1.6 (1.5)	69 (68, 70)	3.0 (1.72)	60 (59, 61)
Grammar	3.8 (1.8)	79 (77, 81)	1.2 (1.6)	69 (67, 70)	3.2 (1.8)	60 (58, 62)
Private	3.8 (1.8)	79 (77, 81)	1.5 (1.7)	69 (67, 71)	3.8 (1.9)	61 (58, 63)
Secondary modern	2.9 (1.7)	78 (76, 80)	1.5 (1.5)	69 (68, 70)	2.0 (1.8)	59 (57, 60)
Other	(ref)	75 (72, 78)	(ref)	67 (64, 70)	(ref)	57 (53, 60)
***Highest educational qualifications***						
None	−7.0 (1.4)	72 (70, 74)	−4.1 (1.2)	66 (64, 67)	−4.4 (1.4)	55 (54, 57)
NVQ1 level/low-grade GCSE/O-levels or equivalent	−1.7 (1.4)	77 (76, 79)	−1.9 (1.2)	68 (66, 69)	−0.4 (1.4)	59 (57, 61)
O-level A-C grade/NVQ3 level or equivalent	− 0.1 (1.3)	79 (77, 81)	− 0.4 (1.1)	69 (68, 71)	0.6 (1.3)	60 (59, 62)
A-levels/NQ3 level or equivalent	0.4 (1.3)	80 (78, 81)	−0.4 (1.1)	69 (68, 71)	0.7 (1.3)	60 (59, 62)
Degree/NVQ4 level or equivalent	−0.1 (1.1)	79 (77, 81)	0.1 (1.0)	70 (69, 71)	0.6 (1.2)	60 (59, 62)
Higher degree/NVQ5 level or equivalent	(ref)	79 (77, 82)	(ref)	70 (68, 72)	(ref)	60 (57, 62)
***Benefits (number of times received)***						
0	17.6 (1.5)	86 (84, 87)	12.0 (1.3)	74 (73, 75)	17.4 (1.6)	67 (65, 68)
1	15.3 (1.6)	83 (82, 85)	10.0 (1.3)	72 (71, 73)	15.3 (1.6)	65 (63, 66)
2	11.9 (1.6)	80 (78, 82)	8.4 (1.4)	70 (69, 72)	13.0 (1.7)	62 (61, 64)
3	3.2 (1.8)	71 (69, 74)	2.8 (1.5)	65 (63, 67)	3.5 (1.8)	53 (51, 55)
4 or more	(ref)	68 (65, 71)	(ref)	62 (59, 65)	(ref)	49 (46, 52)
***Unemployment episodes (number of)***						
0	1.1 (0.7)	79 (77, 80)	0.8 (0.6)	69 (68, 71)	1.9 (0.7)	61 (60, 62)
1	−0.7 (0.8)	77 (75, 79)	−0.9 (0.7)	68 (66, 69)	−1.0 (0.8)	58 (57, 60)
2	−0.1 (1.0)	78 (76, 79)	0.0 (0.8)	69 (67, 70)	−0.5 (1.0)	59 (57, 61)
3 or more	(ref)	78 (76, 79)	(ref)	69 (67, 70)	(ref)	59 (57, 61)
***Age left full-time continuous education***	0.4 (0.1)	–	−0.005 (0.1)	–	0.3 (0.1)	–
***Family income per week (net)***	0.01 (0.01)	–	0.02 (0.004)^a^	–	0.02 (0.01)^a^	–
***Savings and Investments (£1000’s)***	1E-05 (0.0003)^a^	–	0.0004 (0.0002)^a^	–	0.0002 (0.0003)^a^	–
***Debt (£1000’s)***	−0.1 (0.04)	–	−0.02 (0.03)^a^	–	− 0.04 (0.04)^a^	–

^a^small parameter estimates

Results shown in Table 2 and Table S2 (in Additional File 2): *Physical functioning*, *R*^*2*^ *= 0.039; 95% CI: 0.032–0.048, Emotional well-being, R*^*2*^ *= 0.023; 95% CI: 0.017–0.031; General health, R*^*2*^ *= 0.031; 95% CI: 0.024–0.038*.

Less advantaged paternal social class (in relation to physical and general health outcomes) and experience of family financial hardships in childhood were found to be associated with poorer adult health at age 50. In addition, access to free school meals, a measure of low family income, revealed a strong relationship with adult health outcomes (Table 2). For example, Physical functioning scores were on average 4 points higher among those who came from households who did not qualify for access to free school meals compared with those who did. However, access to household amenities had no substantial influence on adult reported physical or emotional health.

2. ‘Habitus and distinction’ class theory

Results shown in Table 3 and Table S2 (in Additional File 2): *Physical functioning*, *R*^*2*^ *= 0.052; 95% CI: 0.043–0.061, Emotional well-being, R*^*2*^ *= 0.028; 95% CI: 0.021–0.035, General health, R*^*2*^ *= 0.029; 95% CI: 0.022–0.036*.

Cognitive ability was positively associated with increased SF-36 scores at age 50. However, participation (voting) in general elections, religious involvement, and trade unions membership were not associated with any notable differences in the health outcomes when compared to non-participation (Table 3). For example, frequent attendance at religious meetings (weekly or more), had similar effects on the mean SF-36 health scores compared to those who had no religion, or attended only monthly, or rarely/never attended at all. Future career aspiration, TV watching, tabloid versus broadsheet newspaper readership, or frequency of book readership were not associated with differences in mean health outcome scores.

3. ‘Exploitation and domination’ class theory

Results shown in Table 4 and Table S2 (in Additional File 2): *Physical functioning*, *R*^*2*^ *= 0.060; 95% CI: 0.051–0.070, Emotional well-being, R*^*2*^ *= 0.052; 95% CI: 0.044–0.062, General health, R*^*2*^ *= 0.058; 95% CI: 0.048–0.067*.

For this social class theory, the most notable effects were observed in relation to housing tenure and household size. Those who lived in larger homes (i.e. 4 or more rooms, excluding bathroom and kitchen) were associated with higher health scores compared to those in small dwellings (0 or 1 room). However, comparison of the mean scores revealed that these differences were not substantial (on average a difference in means of less than 3 points and overlapping 95% CIs). In addition, adjusting for all other factors, the analyses showed that for housing tenure, those owning property (either outright or with help of mortgage) had slightly better General health compared to those renting or squatting.

4. ‘Location within market relations’ social theory

Results shown in Table 5 and Table S2 (in Additional File 2): *Physical functioning*, *R*^*2*^ *= 0.104; 95% CI: 0.092–0.117, Emotional well-being, R*^*2*^ *= 0.067; 95% CI: 0.057–0.077; General health, R*^*2*^ *= 0.083; 95% CI: 0.072–0.094*.

Own social class, educational attainment, and receipt of social security benefits were all associated with the three health outcomes in expected ways. For example: higher social class position (I/II) was associated with better adult health compared to the lower social class position (IV or V/other), having an educational qualification was associated with better health compared to those with no such qualification, and those frequently in receipt of means-tested state benefits had on average worse health outcomes than those who were not. With regard to the latter, particularly large differences in mean health outcomes (12 points or more) were observed between cohort members who did not receive any benefits compared to those in receipt 4 times or more times in adulthood (Table 5).

## Discussion

In this study, it was possible to map NCDS variables to different mechanisms about how social class location is determined and experienced. However, this was a pragmatic rather than necessarily accurate mapping. Generally, the mapped NCDS variables explained little variation in adult health outcomes for any of the social class mechanisms. Of the different mechanisms explored in this study, ‘*Location within market relations’* explained the most variation. Nonetheless, despite these negative results, some relevant associations were observed in the analyses, some expected (e.g. lower parental social class and worse self-reported health in later life) and some not expected (e.g. no meaningful association between health outcomes and voting participation, used as a measure of empowerment).

There are similarities between some of the findings of this study and those previously reported in the literature. The low levels of variation in the selected health outcomes that were explained have been demonstrated previously. In a study assessing childhood risk factors on adult health using the NCDS, Dibben and colleagues were only able to explain 3% of total variation in the SF-36 mental score in their linear regression analyses [17]. Similarly, analyses of other health surveys utilising the SF-36 measures also reported that a relatively small percentage of total variation in the outcomes was explained in their regressions models [5, 22, 23, 37, 41]. Potential reasons for this are discussed further below. This research found that various socio-economic disadvantages in childhood had negative effects on reported health in adulthood: this is consistent with the previous research [2, 14, 33].

However, other research has also suggested that participation in political or other social engagements (e.g. voting, trades unions, religious meetings) can have positive effects on adult health [1, 8] – findings not replicated here. This may in part be because we have used these variables as markers of different kinds of social participation rather than as means of ranking or sorting the population into groups. Other factors (e.g. ethnicity, attitudes, working patterns) have been cited as having important influences on these types of participation [10] and these factors were not explored in our analyses as they were not consistently measured.

### Strengths and weaknesses

This study incorporated a wide range of measures, with some variables created from a combination of information collected across different survey waves in order to maximise use of available information within the NCDS. Nonetheless, for some measures information was missing. Inverse probability weighting and multiple imputation were employed to deal with missing data and thereby minimise potential bias in our findings. In addition, to further validate the SF-36 measures (i.e. to ensure they are showing the expected association with other health measures) we included additional analyses of the three SF-36 health outcomes in relation to both disease-specific measures and general self-assessment of health. The results showed expected associations.

There were limitations to our study that should be considered when interpreting these findings. The range of measures for each of the social class mechanisms was somewhat limited in their coverage (e.g. we did not have any variable to map to ‘power relations’ and we had very limited measures of ‘discrimination’). Even for those mechanisms that we could map, these are unlikely to fully and accurately portray the true exposure to those aspects of social class. We decided to incorporate the measures we had on ‘social closure and opportunity hoarding’, particularly the education measures, into ‘location within market relations’. Arguably these could also have been incorporated into ‘domination and exploitation’ or analysed separately as their own social class theory. Second, the health outcomes were self-reported. As with other measures, self-reported data contain potential sources of bias. Recorded deaths in this cohort are still very low, hence we could not use mortality as an outcome variable, and we were restricted to self-assessed measures. Although SF-36 captures a wide range of health measures suitable for use in population-based studies [11], its validity as a long-term health assessment health tool is still questionable [9, 30]. Consequently, it is important not to over-interpret small differences in results for the various proposed measures of social class theory reported here. In addition, the presence of ceiling scores (particularly for Physical functioning domain) could potentially distort the underlying true patterns of associations of the health experiences of this study population based on these outcomes [11]. It may be that in seeking to explain the differences in health outcomes within the population, rather than us trying to explain differences in outcomes between social groups, we encountered too much random variation that would be expected in essentially attempting to explain differences between individuals (as highlighted by [16]).

### Study implications

The implications of this work are multiple. The key finding – that so little of the variation in outcomes was explained by the different sets of social class variables – suggests different potential explanations. First, it may be that we did not have adequate measures of the social class mechanisms within our dataset to explain the differences in health outcomes. Second, by focusing on explaining variation within a population rather than between social groups, our outcomes were largely determined by random variation and thereby not amenable to this approach. Third, that there is little association between the different social class mechanisms and health outcomes across populations. This is clearly not the case, given the wealth of evidence linking different aspects of social class to different health outcomes, including mortality [4, 13, 18, 40]; however, clearly there is stronger evidence for some mechanisms than others. Fourth, the complex linkages between the different mechanisms (as shown in Fig. 1) means that a different analytical approach is required to better reflect that multiplicity of factors. Fifth, the relatively young age of cohort members may be a factor: clearer effects on health outcomes may be observed in future waves of data. Finally, the ‘disconnect’ between the age at which some exposure variables were measured and the age at which the outcomes were measured may also be relevant.

This study has therefore demonstrated both the potential and the limitations of available cohort studies in measuring aspects of social class theory. This is important when it comes to the design of future studies: we need better ways of accurately capturing different experiences of social class, and of analysing them in relation to appropriate outcomes measured at significant points across the life-course. Further research could usefully include: a follow-up study using the same dataset when there are sufficient deaths to analyse mortality outcomes; replication of the study using other cohort studies, and in different countries and contexts; the development and use of better measures for all social class mechanisms, but in particular the power relations theory and location within market relations (especially with a finer grained consideration of job strain and differentiation within the ‘skilled non-manual’ group). The implications for policy include the need to understand the complex, multifaceted, nature of social and health inequalities, and the associated need for a range of appropriate interventions at different levels and in different spheres: for example, the need for a specific focus on early years’ aspects of class (social background) alongside income-related measures (redistribution).

## Conclusions

Our findings suggest that measures within the NCDS can be mapped to different measures of social class theory linked to health and health inequalities to a certain degree. However, the relatively small amount of variation explained in the outcome variables suggests that these are imperfect measures of the different social class mechanisms. The study lays an important foundation for further research to understand the complex interactions between different aspects of social class and subsequent health outcomes. In doing so, it also emphasises the need to develop and implement improved survey-based, and other, measures to better capture the intricacies of these social mechanisms.

## Supplementary information

**Additional file 1: Table A1.** Results of complete case regression modelling of SF-36 outcomes for each social class theory. **Table A2.** (a): ‘*Social background and early life circumstances*’ class theory: least-squares means of SF-36 outcomes from linear regression. **Table A2.** (b): ‘*Habitus and distinction*’ class theory: least-squares means of SF-36 outcomes from linear regression. **Table A2.** (c): ‘*Exploitation and domination*’ class theory: least-squares means of SF-36 outcomes from linear regression. **Table A2.** (d): ‘*Location within market relations*’: least-squares means of SF-36 outcomes from linear regression.

**Additional file 2: Figure S1.** Mean SF-36 scores by cohort members’ self-rated health within the NCDS measured at age 50. Error bars indicate 95% confidence intervals. **Table S1.** Unadjusted mean SF-36 scores by health problem or condition self-reported by cohort members within the NCDS measured at age 50. CM = Cohort Member; SD = Standard Deviation; Lower, Upper = 95% confidence intervals; MH = Mental Health; Doc = Doctor. **Table S2.** Results of multiply imputed and weighted regression modelling of SF-36 outcomes for each social class theory.

## Data Availability

The datasets generated and/or analysed during the current study are available in the UK Data Service repository, [https://www.ukdataservice.ac.uk]^[2000032]^ .

## Abbreviations

A-level: Advanced Level
CM: Cohort Member
CSE: Certificate of Secondary Education
GCSE: General Certificate of Secondary Education
LS-Means: Least-squares means
NCDS: National Child Development Study
NQV: National Vocational Qualification
NS-SEC: National Statistics Socio-Economic Classification
O-level: Ordinary Level
PSM: Perinatal Mortality Survey
*R*^*2*^: R-squared, the proportion of total variation that is explained by the regression
SC: Social Class
SD: Standard Deviation
SE: Standard Error
SF-36: Short Form-36 Health Survey Questionnaire
TV: Television
95% CI: 95% Confidence Intervals

## Footnotes

1 ***NCDS sample sizes over time:*** 1958 (birth), *N* = 17,415; 1965 (age 7), *N* = 15,425; 1969 (age 11), *N* = 15,337; 1974 (age 16), *N* = 14,654; 1981 (age 23), *N* = 12,537; 1991 (age 33), *N* = 11,469; 2000 (age 42), *N* = 11,419; 2003 (age 45), *N* = 9534; 2004 (age 46), *N* = 9534; 2008 (age 50), *N* = 9790; 2013 (age 55), *N* = 9137.
