# Supplementary material for: Theory driven analysis of social class and health outcomes using UK nationally representative longitudinal data
Source: Int J Equity Health. 2020 Oct 28;19:193. doi: 10.1186/s12939-020-01302-4 (PMC7594287; doi:10.1186/s12939-020-01302-4)
Supplement: Supplementary file 1 — Additional file 1: Table A1. Results of complete case regression modelling of SF-36 outcomes for each social class theory. Table A2. (a): ‘Social background and early life circumstances’ class theory: least-squares means of SF-36 outcomes from linear regression. Table A2. (b): ‘Habitus and distinction’ class theory: least-squares means of SF-36 outcomes from linear regression. Table A2. (c): ‘Exploitation and domination’ class theory: least-squares means of SF-36 outcomes from linear regression. Table A2. (d): ‘Location within market relations’: least-squares means of SF-36 outcomes from linear regression. [file 12939_2020_1302_MOESM1_ESM.docx]

**Table A1: Results of complete case regression modelling of SF-36 outcomes for each social class theory.**

| **(*1). Social background and early life circumstances*** |  | **Physical functioning**  **(N=5,920,** $\boldsymbol{R}^{\boldsymbol{2}}$**=0.040)** | | **Emotional well-being**  **(N=5,917,** $\boldsymbol{R}^{\boldsymbol{2}}$**=0.024)** | | **General health**  **(N=5,933,** $\boldsymbol{R}^{\boldsymbol{2}}$**=0.032)** | |
| --- | --- | --- | --- | --- | --- | --- | --- |
| **Source** | **DF** | **F-Value** | ***p*** | **F-Value** | ***p*** | **F-Value** | ***p*** |
| Gender | 1 | 35.54 | <.001* | 44.75 | <.001* | 2.79 | 0.095 |
| Father's social class | 5 | 5.18 | <.001* | 2.00 | 0.075 | 5.47 | <.001* |
| Grandparent's social class | 5 | 0.21 | 0.960 | 1.46 | 0.201 | 1.66 | 0.141 |
| Mother's education | 3 | 2.29 | 0.076 | 1.81 | 0.143 | 3.49 | 0.015* |
| Father's education | 3 | 3.27 | 0.020* | 0.39 | 0.757 | 1.18 | 0.316 |
| Access to free school meals | 1 | 3.77 | 0.052 | 1.26 | 0.261 | 1.18 | 0.278 |
| Family financial hardships | 2 | 4.49 | 0.011* | 8.27 | <.001* | 9.34 | <.001* |
| Over-crowdedness in childhood | 3 | 4.34 | 0.005* | 1.73 | 0.158 | 0.65 | 0.586 |
| Access to household amenities | 3 | 2.15 | 0.091 | 1.06 | 0.365 | 1.89 | 0.129 |
| ***(2). Habitus and distinction*** |  | **Physical functioning**  **(N=4,312,** $\boldsymbol{R}^{\boldsymbol{2}}$**=0.061)** | | **Emotional well-being**  **(N=4,307,** $\boldsymbol{R}^{\boldsymbol{2}}$**=0.031)** | | **General health**  **(N=4,316,** $\boldsymbol{R}^{\boldsymbol{2}}$**=0.032)** | |
| **Source** | **DF** | **F-Value** | ***p*** | **F-Value** | ***p*** | **F-Value** | ***p*** |
| Gender | 1 | 44.52 | <.001* | 32.83 | <.001* | 0.09 | 0.768 |
| Cognitive Ability (Draw-A-Man Test score) | 1 | 16.08 | <.001* | 4.25 | 0.039* | 6.26 | 0.012* |
| Number of leisure activities in childhood | 5 | 1.25 | 0.282 | 3.45 | 0.004* | 1.11 | 0.351 |
| Imagined occupation at 25 | 7 | 1.89 | 0.067 | 2.32 | 0.023* | 0.95 | 0.465 |
| Aspirations/plans after leaving secondary school | 3 | 4.51 | 0.004* | 2.19 | 0.087 | 2.51 | 0.057 |
| Teachers’ view: Child poor speech rating | 3 | 5.09 | 0.002* | 0.80 | 0.494 | 0.94 | 0.422 |
| Teachers Ability ratings-Maths & English | 1 | 17.21 | <.001* | 0.02 | 0.892 | 8.75 | 0.003* |
| TV watching | 5 | 2.11 | 0.062 | 3.00 | 0.010* | 3.21 | 0.007* |
| Book Readership | 5 | 2.73 | 0.018* | 0.56 | 0.734 | 1.72 | 0.126 |
| Newspaper readership | 3 | 1.74 | 0.157 | 0.44 | 0.723 | 2.41 | 0.065 |
| Voted in general election | 4 | 2.10 | 0.079 | 0.82 | 0.510 | 0.21 | 0.932 |
| Voting intentions | 6 | 2.00 | 0.062 | 1.03 | 0.403 | 2.46 | 0.023* |
| Trades union membership | 1 | 0.00 | 0.949 | 0.16 | 0.690 | 1.55 | 0.213 |
| Attendance at religious meetings | 4 | 1.33 | 0.255 | 0.89 | 0.467 | 2.94 | 0.019* |
| ***(3). Exploitation and domination*** |  | **Physical functioning**  **(N=1,826,** $\boldsymbol{R}^{\boldsymbol{2}}$**=0.024)** | | **Emotional well-being**  **(N=1,825,** $\boldsymbol{R}^{\boldsymbol{2}}$**=0.026)** | | **General health**  **(N=1,828,** $\boldsymbol{R}^{\boldsymbol{2}}$**=0.029)** | |
| **Source** | **DF** | **F-Value** | ***p*** | **F-Value** | ***p*** | **F-Value** | ***p*** |
| Gender | 1 | 5.46 | 0.020* | 8.43 | 0.004* | 1.77 | 0.183 |
| Capital Accrued (1-least to 5-most) | 4 | 0.45 | 0.773 | 3.56 | 0.007* | 2.02 | 0.090 |
| Main finance to buy home | 2 | 0.83 | 0.434 | 0.94 | 0.389 | 2.01 | 0.134 |
| Main source of property purchase price | 4 | 3.54 | 0.007* | 1.26 | 0.284 | 4.67 | 0.001* |
| No. rooms (apart from the bathroom & kitchen) | 5 | 1.03 | 0.400 | 0.72 | 0.609 | 0.39 | 0.860 |
| Mortgage as percent of house price | 6 | 1.32 | 0.243 | 0.91 | 0.489 | 1.95 | 0.070 |
| Tenure | 5 | 1.22 | 0.298 | 1.44 | 0.207 | 1.73 | 0.125 |
| ***(4). Location within market relations*** |  | **Physical health**  **(N=4,346,** $\boldsymbol{R}^{\boldsymbol{2}}$**=0.091)** | | **Emotional well-being**  **(N=4,347,** $\boldsymbol{R}^{\boldsymbol{2}}$**=0.052)** | | **General health**  **(N=4,352,** $\boldsymbol{R}^{\boldsymbol{2}}$**=0.072)** | |
| **Source** | **DF** | **F-Value** | ***p*** | **F-Value** | ***p*** | **F-Value** | ***p*** |
| Gender | 1 | 15.64 | <.001* | 13.66 | <.001* | 6.94 | 0.008* |
| Social Class | 5 | 5.92 | <.001* | 5.57 | <.001* | 5.05 | <0.001* |
| Type of Secondary School | 4 | 2.37 | 0.050 | 0.87 | 0.481 | 2.08 | 0.081 |
| Highest Qualifications | 5 | 5.46 | <.001* | 3.29 | 0.006* | 3.60 | 0.003* |
| No. of times on benefits | 4 | 30.59 | <.001* | 11.7 | <.001* | 25.93 | <.001* |
| No. of unemployment episodes | 3 | 2.69 | 0.045* | 4.38 | 0.004* | 4.23 | 0.005* |
| Age left full-time continuous education | 1 | 5.06 | 0.025* | 0.04 | 0.840 | 4.72 | 0.030* |
| Family income per week (net) | 1 | 1.44 | 0.230 | 6.31 | 0.012* | 2.60 | 0.107 |
| Savings and Investments (£1000's) | 1 | 0.93 | 0.336 | 2.77 | 0.096 | 0.82 | 0.365 |
| Debt (£1000's) | 1 | 0.24 | 0.625 | 0.62 | 0.431 | 0.59 | 0.443 |

**p*<0.05 (level of significance); *DF=degrees of freedom*

**Table A2 (a): ‘*Social background and early life circumstances*’ class theory: least-squares means of SF-36 outcomes from linear regression.**

|  | **Physical functioning** | | | **Emotional well-being** | | | **General health** | | |
| --- | --- | --- | --- | --- | --- | --- | --- | --- | --- |
| **Variable** | **Mean** | **95% CI** | | **Mean** | **95% CI** | | **Mean** | **95% CI** | |
| ***Gender:*** |  |  |  |  |  |  |  |  |  |
| Female | 83.3 | 80.6 | 86.0 | 71.6 | 69.4 | 73.9 | 66.5 | 63.8 | 69.3 |
| Male | 86.6 | 83.9 | 89.3 | 74.7 | 72.4 | 77.0 | 65.6 | 62.9 | 68.3 |
| ***Father's Social Class:*** |  |  |  |  |  |  |  |  |  |
| Professional (I) | 87.4 | 84.1 | 90.8 | 74.1 | 71.3 | 76.9 | 69.4 | 66.0 | 72.9 |
| Managerial-technical (II) | 86.7 | 83.7 | 89.6 | 73.4 | 70.9 | 75.9 | 67.4 | 64.4 | 70.3 |
| Skilled non-manual (III-NM) | 86.8 | 83.6 | 89.9 | 74.2 | 71.5 | 76.8 | 67.7 | 64.5 | 71.0 |
| Skilled-manual (III-M) | 84.3 | 81.4 | 87.1 | 72.6 | 70.2 | 75.0 | 65.4 | 62.5 | 68.2 |
| Partly skilled (IV) | 83.2 | 80.2 | 86.1 | 73.6 | 71.1 | 76.0 | 64.7 | 61.8 | 67.7 |
| Unskilled & Other (V/Other) | 81.3 | 78.1 | 84.5 | 71.1 | 68.4 | 73.8 | 61.7 | 58.5 | 65.0 |
| ***Grandparent’s Social Class:*** |  |  |  |  |  |  |  |  |  |
| Professional (I) | 83.99 | 79.8 | 88.2 | 73.1 | 69.5 | 76.7 | 64.8 | 60.4 | 69.1 |
| Managerial-technical (II) | 84.77 | 81.9 | 87.7 | 74.2 | 71.8 | 76.7 | 67.0 | 64.0 | 69.9 |
| Skilled non-manual (III-NM) | 85.72 | 82.5 | 88.9 | 73.6 | 70.9 | 76.3 | 66.7 | 63.5 | 70.0 |
| Skilled-manual (III-M) | 84.91 | 82.2 | 87.6 | 72.3 | 70.0 | 74.6 | 65.1 | 62.3 | 67.9 |
| Partly skilled (IV) | 84.94 | 82.1 | 87.8 | 72.8 | 70.4 | 75.2 | 65.7 | 62.8 | 68.6 |
| Unskilled & Other (V/Other) | 85.24 | 82.1 | 88.4 | 73.0 | 70.3 | 75.6 | 67.1 | 63.9 | 70.3 |
| ***Mother's education (Did stay at school after min. leaving age?):*** |  |  |  |  |  |  |  |  |  |
| Did not stay-16 years | 83.7 | 81.1 | 86.3 | 72.3 | 70.1 | 74.6 | 64.8 | 62.1 | 67.5 |
| Did stay-16 years | 84.6 | 81.6 | 87.6 | 74.1 | 716 | 76.7 | 65.7 | 62.6 | 68.8 |
| Did stay-16 to 18 years | 85.9 | 83.0 | 88.7 | 72.8 | 70.4 | 75.2 | 67.6 | 64.7 | 70.5 |
| Did stay-19 or more years | 85.6 | 81.6 | 89.6 | 73.4 | 70.0 | 76.7 | 66.1 | 62.0 | 70.2 |
| ***Father's education (Did stay at school after min. leaving age?):*** |  |  |  |  |  |  |  |  |  |
| Did not stay-16 years | 83.4 | 80.7 | 86.1 | 73.1 | 70.8 | 75.4 | 65.0 | 62.2 | 67.7 |
| Did stay-16 years | 84.1 | 81.0 | 87.2 | 73.1 | 70.5 | 75.7 | 66.0 | 62.9 | 69.1 |
| Did stay-16 to 18 years | 86.2 | 83.2 | 89.2 | 73.8 | 71.3 | 76.3 | 66.5 | 63.5 | 69.5 |
| Did stay-19 or more years | 86.1 | 82.4 | 89.7 | 72.6 | 69.5 | 75.7 | 66.8 | 63.0 | 70.5 |
| ***Receipt of free school meals by any child in the household:*** |  |  |  |  |  |  |  |  |  |
| No | 85.9 | 83.1 | 88.7 | 73.6 | 71.3 | 76.0 | 66.6 | 63.8 | 69.4 |
| Yes | 84.0 | 81.1 | 86.8 | 72.9 | 70.3 | 75.1 | 65.5 | 62.6 | 68.4 |
| ***Experienced family financial hardships in childhood:*** |  |  |  |  |  |  |  |  |  |
| Never | 86.5 | 83.8 | 89.1 | 75.5 | 73.3 | 77.7 | 68.9 | 66.2 | 71.5 |
| Once | 83.8 | 80.9 | 86.7 | 73.6 | 71.2 | 76.1 | 65.4 | 62.4 | 68.3 |
| Twice or more | 84.5 | 80.8 | 88.2 | 70.4 | 67.2 | 73.5 | 64.0 | 60.2 | 67.7 |
| ***Experienced overcrowding in childhood:*** |  |  |  |  |  |  |  |  |  |
| Never | 86.8 | 84.2 | 89.4 | 73.9 | 71. 7 | 76.1 | 66.8 | 64.2 | 69.5 |
| Once | 84.7 | 81.7 | 87.6 | 73.1 | 70.6 | 75.6 | 66.1 | 63.1 | 69.1 |
| Twice | 85.8 | 82.5 | 89.1 | 74.2 | 71.4 | 77.0 | 66.3 | 62.9 | 69.6 |
| Three times or more | 82.5 | 78.9 | 86.0 | 71.4 | 68.4 | 74.4 | 65.1 | 61.4 | 69.0 |
| ***Access to household amenities*:*** |  |  |  |  |  |  |  |  |  |
| Shared or no sole use | 89.5 | 83.5 | 95.4 | 74.0 | 68.9 | 79.0 | 68.3 | 62.2 | 74.3 |
| Sole use of 1 | 79.9 | 74.7 | 85.1 | 70.3 | 65.9 | 74.7 | 61.3 | 56.0 | 66.6 |
| Sole use of 2 | 85.4 | 81.5 | 89.2 | 74.9 | 71.7 | 78.1 | 67.3 | 63.4 | 71.2 |
| Sole use of 3 | 85.0 | 83.3 | 86.7 | 73.4 | 72.0 | 74.8 | 67.4 | 65.7 | 69.0 |

**Access to household amenities: indoor toilet, bathroom, kitchen.*

**Table A2 (b): ‘*Habitus and distinction*’ class theory: least-squares means of SF-36 outcomes from linear regression.**

|  | **Physical functioning** | | | **Emotional well-being** | | | **General health** | | |
| --- | --- | --- | --- | --- | --- | --- | --- | --- | --- |
|  |  | | |  | | |  | | |
| **Variable** | **Mean** | **95% CI** | | **Mean** | **95% CI** | | **Mean** | **95% CI** | |
| ***Gender:*** |  |  |  |  |  |  |  |  |  |
| Female | 78.9 | 69.6 | 88.1 | 66.4 | 58.3 | 74.5 | 70.4 | 60.7 | 80.0 |
| Male | 83.7 | 74.5 | 93.0 | 70.1 | 62.0 | 78.1 | 70.6 | 61.0 | 80.2 |
| ***Number of leisure time activities in childhood:*** |  |  |  |  |  |  |  |  |  |
| 0 | 90.7 | 50.5 | 100.0* | 52.3 | 17.2 | 87.3 | 64.3 | 22.6 | 100.0* |
| 1 | 70.3 | 56.9 | 83.6 | 61.1 | 49.4 | 72.7 | 68.5 | 54.6 | 82.3 |
| 2 | 83.1 | 75.0 | 91.1 | 70.7 | 63.7 | 77.7 | 77.2 | 68.9 | 85.6 |
| 3 | 81.5 | 74.9 | 88.1 | 75.0 | 69.2 | 80.8 | 71.1 | 64.3 | 78.0 |
| 4 | 80.5 | 74.2 | 86.7 | 74.4 | 68.9 | 79.8 | 70.5 | 64.0 | 77.0 |
| 5 or more | 82.0 | 76.0 | 88.0 | 76.0 | 70.7 | 81.2 | 71.3 | 65.1 | 77.5 |
| ***Imagined occupation at age 25:*** |  |  |  |  |  |  |  |  |  |
| Higher managerial & professional | 80.6 | 71.3 | 89.9 | 65.0 | 56.9 | 73.2 | 68.7 | 59.1 | 78.4 |
| Lower managerial & professional | 82.0 | 72.8 | 91.1 | 67.5 | 59.5 | 75.5 | 71.6 | 62.0 | 81.1 |
| Intermediate occupations | 80.9 | 71.7 | 90.1 | 68.1 | 60.1 | 76.2 | 70.9 | 61.3 | 80.5 |
| Small employers & Own account workers | 83.2 | 69.2 | 97.3 | 73.0 | 60.5 | 85.5 | 69.5 | 54.9 | 84.1 |
| Lower supervisory & Technical | 80.2 | 70.9 | 89.4 | 68.9 | 60.8 | 77.0 | 70.4 | 60.7 | 80.0 |
| Semi-routine occupations | 80.2 | 71.0 | 89.5 | 66.7 | 58.6 | 74.8 | 70.1 | 60.5 | 79.8 |
| Routine | 84.4 | 74.8 | 93.9 | 69.3 | 60.9 | 77.6 | 72.0 | 62.1 | 82.0 |
| Students/Not stated/Inadequately described/Not classified | 79.1 | 69.9 | 88.3 | 67.3 | 59.3 | 75.3 | 70.6 | 61.0 | 80.2 |
| ***Aspiration after secondary school:*** |  |  |  |  |  |  |  |  |  |
| Full time studies | 82.2 | 72.9 | 91.4 | 68.9 | 60.8 | 77.0 | 71.4 | 61.8 | 81.0 |
| Job, nothing more | 79.9 | 70.6 | 89.2 | 68.4 | 60.3 | 76.5 | 69.2 | 59.5 | 78.8 |
| Part study, job | 82.8 | 73.5 | 92.1 | 68.8 | 60.7 | 76.9 | 71.5 | 61.8 | 81.1 |
| Don't know | 80.3 | 71.0 | 89.6 | 66.8 | 58.7 | 74.9 | 69.9 | 60.2 | 79.5 |
| ***Teachers' rating: child's poor speech:*** |  |  |  |  |  |  |  |  |  |
| Certainly | 63.4 | 48.8 | 78.0 | 61.1 | 48.4 | 73.9 | 79.5 | 64.3 | 94.7 |
| Not at all | 87.0 | 79.7 | 94.4 | 68.9 | 62.4 | 75.3 | 69.0 | 61.4 | 76.7 |
| Somewhat | 85.1 | 77.2 | 93.0 | 68.7 | 61.8 | 75.6 | 69.4 | 61.2 | 77.6 |
| Don’t know | 89.7 | 70.5 | 100.0* | 74.2 | 57.4 | 91.0 | 64.0 | 44.1 | 84.0 |
| ***Teachers' ability rating: Maths & English:*** |  |  |  |  |  |  |  |  |  |
| Little ability/below average CSE grades 2-4/O-level/CSE 1 | 79.6 | 70.4 | 88.8 | 68.3 | 60.2 | 76.3 | 69.2 | 59.6 | 78.8 |
| A-level and higher | 83.0 | 73.7 | 92.3 | 68.2 | 60.1 | 76.3 | 71.8 | 62.1 | 81.4 |
| ***TV watching:*** |  |  |  |  |  |  |  |  |  |
| 1-2 times a week | 82.3 | 73.1 | 91.5 | 70.6 | 62.5 | 78.6 | 71.2 | 61.7 | 80.8 |
| 3-4 times a week | 82.7 | 73.5 | 91.9 | 69.7 | 61.7 | 77.8 | 72.3 | 62.7 | 81.9 |
| 5 times a week + | 80.7 | 71.6 | 89.9 | 67.8 | 59.9 | 75.8 | 70.0 | 60.6 | 79.5 |
| One in last 4 weeks | 75.6 | 63.9 | 87.2 | 66.7 | 56.6 | 76.8 | 61.9 | 49.9 | 73.9 |
| 2-3 times in last 4 weeks | 82.6 | 73.0 | 92.3 | 67.1 | 58.7 | 75.5 | 73.1 | 63.1 | 83.1 |
| Not done in last 4 weeks | 84.0 | 73.3 | 94.6 | 67.5 | 58.2 | 76.8 | 74.3 | 63.3 | 85.4 |
| ***Book readership:*** |  |  |  |  |  |  |  |  |  |
| 1-2 times a week | 81.8 | 72.4 | 91.2 | 68.2 | 60.0 | 76.4 | 71.3 | 61.5 | 81.0 |
| 3-4 times a week | 81.5 | 72.2 | 90.9 | 67.8 | 59.6 | 76.0 | 69.1 | 59.4 | 78.8 |
| 5 times a week + | 82.1 | 72.8 | 91.4 | 68.7 | 60.6 | 76.8 | 70.5 | 60.9 | 80.1 |
| One in last 4 weeks | 81.7 | 72.3 | 91.0 | 67.6 | 59.4 | 75.7 | 72.3 | 62.6 | 82.0 |
| 2-3 times in last 4 weeks | 81.5 | 72.0 | 90.9 | 69.2 | 60.9 | 77.4 | 70.2 | 60.3 | 80.0 |
| Not done in last 4 weeks | 79.3 | 70.0 | 88.5 | 68.0 | 59.9 | 76.1 | 69.6 | 59.9 | 79.2 |
| ***Newspaper readership:*** |  |  |  |  |  |  |  |  |  |
| Tabloid (Daily papers-Express, Mail, Star, Evening; Mirror, Sun) | 81.5 | 72.2 | 90.7 | 67.9 | 59.8 | 75.9 | 70.1 | 60.5 | 79.7 |
| Broadsheet (Telegraph, Fin. Times, Guardian, Times) | 82.9 | 73.5 | 92.3 | 68.9 | 60.7 | 77.1 | 71.6 | 61.9 | 81.4 |
| Other (e.g. Newsline, Regional etc) | 80.6 | 71.3 | 89.9 | 68.3 | 60.2 | 76.5 | 71.4 | 61.7 | 81.1 |
| None | 80.3 | 71.0 | 89.6 | 67.9 | 59.8 | 76.0 | 68.8 | 59.1 | 78.5 |
| ***Voting behaviour (party voted for in last general elections):*** |  |  |  |  |  |  |  |  |  |
| Conservative | 82.9 | 73.6 | 92.2 | 68.2 | 60.0 | 76.3 | 71.1 | 61.4 | 80.7 |
| Labour | 81.7 | 72.4 | 90.9 | 68.0 | 59.9 | 76.1 | 70.3 | 60.7 | 79.9 |
| Liberal | 83.0 | 73.6 | 92.5 | 68.9 | 60.7 | 77.1 | 70.8 | 61.0 | 80.6 |
| Other (Welsh Nat, Scots Nat, Nat front, Communist, WRP) | 76.9 | 67.0 | 86.9 | 68.9 | 60.3 | 77.6 | 69.5 | 59.2 | 79.8 |
| None | 82.0 | 72.7 | 91.3 | 67.2 | 59.1 | 75.3 | 70.7 | 61.1 | 80.4 |
| ***Current participation (current voting intentions):*** |  |  |  |  |  |  |  |  |  |
| Conservative | 82.8 | 73.5 | 92.1 | 69.9 | 61.7 | 78.0 | 72.8 | 63.1 | 82.5 |
| Labour | 80.8 | 71.5 | 90.1 | 67.9 | 59.8 | 76.0 | 70.1 | 60.4 | 79.7 |
| Liberal | 83.0 | 73.6 | 92.4 | 68.8 | 60.6 | 77.0 | 72.3 | 62.6 | 82.1 |
| Social DP | 80.7 | 71.4 | 90.0 | 68.3 | 60.2 | 76.4 | 70.3 | 60.6 | 79.9 |
| Other | 77.9 | 68.0 | 87.7 | 66.3 | 57.7 | 74.9 | 65.8 | 55.6 | 76.1 |
| None/Spoil | 80.8 | 71.4 | 90.2 | 68.5 | 60.3 | 76.7 | 70.1 | 60.4 | 79.9 |
| Refused/Don't Know | 83.2 | 73.7 | 92.7 | 68.0 | 59.7 | 76.3 | 72.0 | 62.1 | 81.8 |
| ***Trades union membership:*** |  |  |  |  |  |  |  |  |  |
| No | 81.3 | 72.1 | 90.5 | 68.3 | 60.3 | 76.4 | 71.0 | 61.3 | 80.5 |
| Yes | 81.3 | 72.1 | 90.6 | 68.1 | 60.1 | 76.2 | 70.1 | 60.5 | 79.7 |
| ***Religion (attendance at religious meetings):*** |  |  |  |  |  |  |  |  |  |
| Weekly or More | 80.2 | 70.8 | 89.6 | 67.6 | 59.4 | 75.8 | 68.5 | 58.8 | 78.3 |
| Monthly or More | 83.1 | 73.4 | 92.8 | 68.2 | 59.7 | 76.7 | 75.1 | 65.0 | 85.2 |
| Less than monthly | 80.8 | 71.4 | 90.2 | 69.5 | 61.3 | 77.7 | 69.6 | 59.9 | 79.4 |
| Rarely or Never | 81.8 | 72.6 | 91.1 | 67.8 | 59.7 | 75.9 | 69.7 | 60.1 | 79.3 |
| No religion | 80.6 | 71.4 | 89.8 | 68.0 | 60.0 | 76.1 | 69.5 | 59.9 | 79.0 |

**Rounded-off to the maximum achievable score.*

**Table A2 (c): ‘*Exploitation and domination*’ class theory: least-squares means of SF-36 outcomes from linear regression.**

|  | **Physical functioning** | | | **Emotional Well-Being** | | | **General health** | | |
| --- | --- | --- | --- | --- | --- | --- | --- | --- | --- |
| **Variable** | **Mean** | **95% CI** | | **Mean** | **95% CI** | | **Mean** | **95% CI** | |
| ***Gender:*** |  |  |  |  |  |  |  |  |  |
| Female | 81.8 | 73.9 | 89.6 | 71.8 | 64.3 | 79.2 | 60.5 | 51.7 | 69.3 |
| Male | 83.8 | 75.9 | 91.7 | 74.1 | 66.7 | 81.6 | 59.3 | 50.5 | 68.1 |
| ***Housing tenure:*** |  |  |  |  |  |  |  |  |  |
| Own - outright | 82.8 | 76.8 | 88.9 | 76.5 | 70.7 | 82.2 | 65.1 | 58.3 | 71.9 |
| Own - buying with help of mortgage/loan | 83.1 | 77.0 | 89.1 | 76.3 | 70.6 | 82.0 | 65.2 | 58.5 | 72.0 |
| Rent it | 77.5 | 69.9 | 85.0 | 71.6 | 64.5 | 78.8 | 58.8 | 50.3 | 67.2 |
| Pay part rent/part mortgage(shared/eq o) | 85.5 | 60.5 | 100.0* | 63.5 | 39.8 | 87.2 | 44.4 | 16.5 | 72.4 |
| Live rent free, incl. in rels/friends prop | 86.8 | 73.2 | 100.0* | 69.9 | 57.0 | 82.7 | 65.9 | 50.7 | 81.1 |
| Other | 80.9 | 67.3 | 94.5 | 79.8 | 67.0 | 92.7 | 59.9 | 44.9 | 75.1 |
| ***Household size (excl. bathroom & kitchen):*** |  |  |  |  |  |  |  |  |  |
| 0 | 82.8 | 68.6 | 97.0 | 68.9 | 55.4 | 82.3 | 63.9 | 48.1 | 79.8 |
| 2 | 88.5 | 74.3 | 102.7 | 76.0 | 62.6 | 89.4 | 60.9 | 45.1 | 76.8 |
| 3 | 79.2 | 71.2 | 87.2 | 72.6 | 65.1 | 80.1 | 58.6 | 49.7 | 67.5 |
| 4 | 81.6 | 74.1 | 89.0 | 73.1 | 66.1 | 80.1 | 59.2 | 50.9 | 67.5 |
| 5 | 81.7 | 74.2 | 89.1 | 72.8 | 65.7 | 79.8 | 58.0 | 49.7 | 66.3 |
| 6 | 82.9 | 75.4 | 90.3 | 74.3 | 67.3 | 81.3 | 58.8 | 50.5 | 67.1 |
| ***Capital accrued (ranks):*** |  |  |  |  |  |  |  |  |  |
| 1 (least) | 81.4 | 73.1 | 89.7 | 72.0 | 64.2 | 79.9 | 57.5 | 48.2 | 66.7 |
| 2 | 82.3 | 74.1 | 90.5 | 70.7 | 63.0 | 78.4 | 57.8 | 48.7 | 66.9 |
| 3 | 83.1 | 75.1 | 91.0 | 73.3 | 65.7 | 80.8 | 60.4 | 51.5 | 69.3 |
| 4 | 83.8 | 75.9 | 91.9 | 75.9 | 68.3 | 83.6 | 62.2 | 53.2 | 71.2 |
| 5 (most) | 83.3 | 75.2 | 91.4 | 72.8 | 65.2 | 80.5 | 61.6 | 52.6 | 70.6 |
| ***Main finance used to by home:*** |  |  |  |  |  |  |  |  |  |
| Building Society | 84.7 | 77.7 | 91.7 | 71.4 | 64.8 | 78.0 | 62.1 | 54.3 | 69.9 |
| Mortgage/Loan | 85.5 | 78.5 | 92.5 | 70.3 | 63.7 | 76.9 | 59.1 | 51.3 | 66.9 |
| Other ways (e.g. Sale property, Savings) | 78.1 | 64.6 | 91.5 | 77.1 | 64.4 | 89.8 | 58.6 | 43.6 | 73.6 |
| ***Type of financial source for home purchase:*** |  |  |  |  |  |  |  |  |  |
| Building Society Mortgage | 88.0 | 81.1 | 94.9 | 76.0 | 69.5 | 82.6 | 67.1 | 59.3 | 74.8 |
| Bank Mortgage/Loan | 89.8 | 82.5 | 97.1 | 77.0 | 70.1 | 83.9 | 70.0 | 61.8 | 78.1 |
| Other Loan( e.g. Private, Govt Homeloan) | 89.0 | 80.9 | 97.1 | 73.1 | 65.4 | 80.8 | 66.0 | 56.9 | 75.1 |
| Gift/Inheritance | 57.7 | 38.8 | 76.6 | 63.3 | 45.4 | 81.2 | 29.8 | 8.7 | 50.9 |
| Other ways (e.g. Sale of previous property, Savings) | 89.3 | 81.6 | 97.0 | 75.3 | 68.0 | 82.6 | 66.6 | 58.1 | 75.2 |
| ***Mortgage as a percent of house price:*** |  |  |  |  |  |  |  |  |  |
| Under 50% | 87.0 | 78.1 | 96.0 | 73.9 | 65.5 | 82.4 | 61.9 | 51.9 | 71.8 |
| 50–60% | 81.4 | 72.5 | 90.3 | 75.2 | 66.8 | 83.7 | 60.1 | 50.2 | 70.1 |
| 60–70% | 82.4 | 73.7 | 91.0 | 72.0 | 63.8 | 80.2 | 59.4 | 49.8 | 69.1 |
| 70–80% | 83.1 | 74.7 | 91.5 | 71.3 | 63.4 | 79.2 | 58.0 | 48.6 | 67.4 |
| 80–90% | 81.8 | 73.7 | 90.0 | 72.0 | 64.3 | 79.7 | 56.9 | 47.8 | 66.0 |
| 90–100% | 83.8 | 75.6 | 92.0 | 73.6 | 65.8 | 81.4 | 61.1 | 52.0 | 70.3 |
| 100% | 79.8 | 70.5 | 89.1 | 72.4 | 63.6 | 81.2 | 61.8 | 51.5 | 72.2 |

**Rounded-off to the maximum achievable score.*

**Table A2 (d): ‘*Location within market relations*’: least-squares means of SF-36 outcomes from linear regression.**

|  | **Physical functioning** | | | **Emotional well-being** | | | **General health** | | |
| --- | --- | --- | --- | --- | --- | --- | --- | --- | --- |
| **Variable** | **Mean** | **95% CI** | | **Mean** | **95% CI** | | **Mean** | **95% CI** | |
| ***Gender:*** |  |  |  |  |  |  |  |  |  |
| Female | 76.2 | 74.0 | 78.3 | 69.8 | 67.9 | 71.7 | 62.5 | 60.2 | 64.7 |
| Male | 78.8 | 76.6 | 81.0 | 71.9 | 70.0 | 73.8 | 60.6 | 58.4 | 62.9 |
| ***Own Social Class:*** |  |  |  |  |  |  |  |  |  |
| Professional (I) | 82.4 | 80.0 | 84.9 | 72.6 | 70.5 | 74.6 | 64.6 | 62.0 | 67.1 |
| Managerial-technical (II) | 80.5 | 78.7 | 82.3 | 72.6 | 71.1 | 74.2 | 63.3 | 61.5 | 65.2 |
| Skilled non-manual (III-NM) | 79.4 | 77.4 | 81.5 | 70.8 | 69.1 | 72.6 | 60.9 | 58.8 | 63.0 |
| Skilled-manual (III-M) | 76.8 | 74.7 | 79.0 | 72.3 | 70.4 | 74.1 | 61.3 | 59.1 | 63.5 |
| Partly skilled (IV) | 74.5 | 71.2 | 77.8 | 65.3 | 62.5 | 68.1 | 55.8 | 52.4 | 59.2 |
| Unskilled & Other (V/Other) | 71.4 | 62.3 | 80.5 | 71.5 | 63.8 | 79.3 | 63.4 | 54.0 | 72.7 |
| ***Type of Secondary School:*** |  |  |  |  |  |  |  |  |  |
| Private | 79.0 | 75.9 | 82.0 | 72.1 | 69.6 | 74.7 | 63.2 | 60.1 | 66.3 |
| Grammar | 79.8 | 77.3 | 82.3 | 70.8 | 68.7 | 73.0 | 63.2 | 60.6 | 65.8 |
| Secondary modern | 77.7 | 75.5 | 79.9 | 70.8 | 68.9 | 72.7 | 60.9 | 58.6 | 63.2 |
| Comprehensive | 77.8 | 75.8 | 79.8 | 71.5 | 69.8 | 73.1 | 62.3 | 60.3 | 64.3 |
| Other | 73.2 | 68.6 | 77.9 | 69.0 | 65.1 | 73.0 | 58.2 | 53.4 | 62.9 |
| ***Highest educational qualifications:*** |  |  |  |  |  |  |  |  |  |
| None | 72.8 | 69.9 | 75.7 | 68.4 | 65.9 | 70.9 | 58.2 | 55.2 | 61.2 |
| NVQ1 level/low-grade GCSE/O-levels or equivalent | 75.9 | 73.2 | 78.6 | 68.9 | 66.6 | 71.2 | 59.6 | 56.9 | 62.4 |
| O-level A-C grade/NVQ3 level or equivalent | 79.0 | 76.6 | 81.3 | 71.4 | 69.4 | 73.3 | 62.9 | 60.5 | 65.2 |
| A-levels/NQ3 level or equivalent | 79.2 | 76.8 | 81.7 | 71.3 | 69.2 | 73.3 | 62.8 | 60.3 | 65.3 |
| Degree/NVQ4 level or equivalent | 78.5 | 76.1 | 80.9 | 71.3 | 69.3 | 73.4 | 62.2 | 59.7 | 64.6 |
| Higher degree/NVQ5 level or equivalent | 79.6 | 75.9 | 83.4 | 73.9 | 70.7 | 77.1 | 63.8 | 59.8 | 67.5 |
| ***Benefits (number of times received):*** |  |  |  |  |  |  |  |  |  |
| 0 | 84.7 | 82.5 | 86.8 | 74.8 | 72.9 | 76.6 | 68.3 | 66.1 | 70.5 |
| 1 | 82.5 | 80.3 | 84.8 | 72.9 | 71.0 | 74.9 | 66.2 | 63.9 | 68.6 |
| 2 | 80.6 | 78.0 | 83.2 | 72.6 | 70.4 | 74.8 | 64.8 | 62.1 | 67.5 |
| 3 | 70.8 | 67.4 | 74.2 | 68.3 | 65.4 | 71.2 | 55.2 | 51.7 | 58.7 |
| 4 or more | 68.9 | 64.6 | 73.1 | 65.7 | 62.1 | 69.3 | 53.3 | 48.95 | 57.6 |
| ***Unemployment episodes (number of):*** |  |  |  |  |  |  |  |  |  |
| 0 | 78.9 | 76.8 | 81.1 | 72.5 | 70.6 | 74.3 | 63.4 | 61.2 | 65.6 |
| 1 | 77.4 | 75.1 | 79.6 | 70.8 | 68.9 | 72.7 | 61.1 | 58.8 | 63.4 |
| 2 | 77.8 | 75.0 | 80.5 | 71.1 | 68.7 | 73.4 | 61.2 | 58.4 | 64.0 |
| 3 or more | 75.9 | 72.8 | 79.1 | 69.1 | 66.4 | 71.8 | 60.5 | 57.3 | 63.7 |
