# Supplementary material for: Theory driven analysis of social class and health outcomes using UK nationally representative longitudinal data
Source: Int J Equity Health. 2020 Oct 28;19:193. doi: 10.1186/s12939-020-01302-4 (PMC7594287; doi:10.1186/s12939-020-01302-4)
Supplement: Supplementary file 2 — Additional file 2: Figure S1. Mean SF-36 scores by cohort members’ self-rated health within the NCDS measured at age 50. Error bars indicate 95% confidence intervals. Table S1. Unadjusted mean SF-36 scores by health problem or condition self-reported by cohort members within the NCDS measured at age 50. CM = Cohort Member; SD = Standard Deviation; Lower, Upper = 95% confidence intervals; MH = Mental Health; Doc = Doctor. Table S2. Results of multiply imputed and weighted regression modelling of SF-36 outcomes for each social class theory. [file 12939_2020_1302_MOESM2_ESM.docx]

**Figure S1: Mean SF-36 scores by cohort members’ self-rated health within the NCDS measured at age 50.** Error bars indicate 95% confidence intervals.


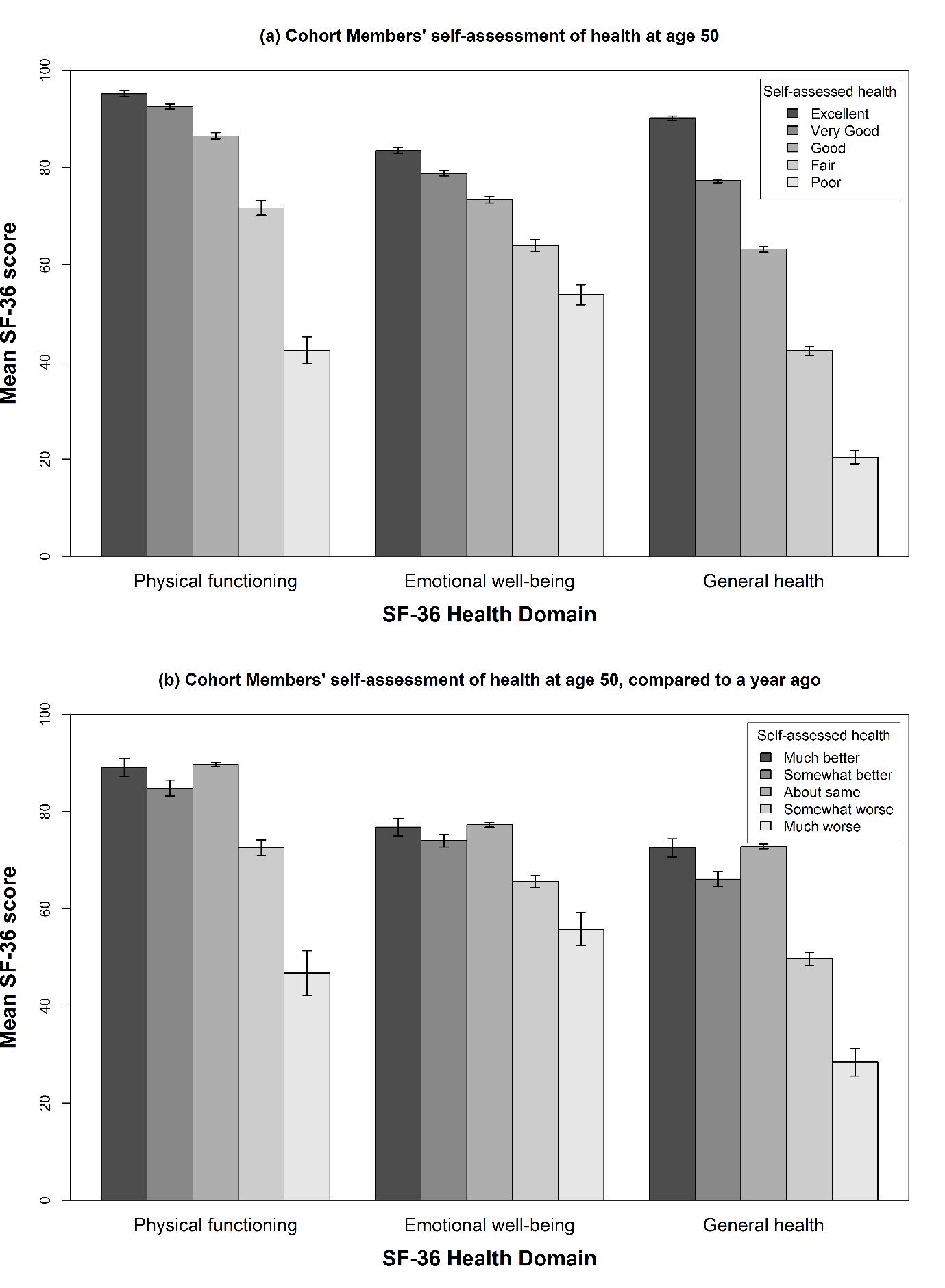


**Table S1: Unadjusted mean SF-36 scores by health problem or condition self-reported by cohort members within the NCDS measured at age 50**. CM=Cohort Member; SD=Standard Deviation; Lower, Upper= 95% confidence intervals; MH=Mental Health; Doc=Doctor.

| **[Questionnaire Variable] Health Problem** | **Survey Response** | **SF-36 Physical functioning score** | | | | | **SF-36 Emotional well-being score** | | | | | **SF-36 General health score** | | | | |
| --- | --- | --- | --- | --- | --- | --- | --- | --- | --- | --- | --- | --- | --- | --- | --- | --- |
|  |  | **n** | **Mean** | **SD** | **Lower** | **Upper** | **n** | **Mean** | **SD** | **Lower** | **Upper** | **n** | **Mean** | **SD** | **Lower** | **Upper** |
| **[KHLPRB1] Whether CM suffers health problems: Asthma or wheezy bronchitis** | Not mentioned | 7,923 | 87.3 | 20.5 | 86.8 | 87.7 | 7,914 | 75.6 | 17.7 | 75.2 | 76.0 | 7,937 | 69.7 | 21.1 | 69.2 | 70.2 |
|  | Mentioned | 841 | 74.8 | 28.4 | 72.9 | 76.8 | 841 | 69.6 | 21.2 | 68.2 | 71.0 | 844 | 55.3 | 25.2 | 53.6 | 57.1 |
|  |  |  |  |  |  |  |  |  |  |  |  |  |  |  |  |  |
| **[KHLPRB3] Whether CM suffers health problems: (sugar) Diabetes** | Not mentioned | 8,390 | 86.7 | 21.0 | 86.3 | 87.2 | 8,380 | 75.2 | 18.0 | 74.8 | 75.6 | 8,405 | 69.4 | 21.4 | 68.9 | 69.8 |
|  | Mentioned | 374 | 71.7 | 30.3 | 68.6 | 74.8 | 375 | 70.5 | 20.3 | 68.5 | 72.6 | 376 | 45.1 | 22.9 | 42.8 | 47.4 |
|  |  |  |  |  |  |  |  |  |  |  |  |  |  |  |  |  |
| **[KHLPRB4] Whether CM suffers health problems: Convulsion, fit, epilepsy** | Not mentioned | 8,700 | 86.3 | 21.4 | 85.8 | 86.7 | 8,691 | 75.1 | 18.0 | 74.8 | 75.5 | 8,717 | 68.5 | 21.8 | 68.1 | 69.0 |
|  | Mentioned | 64 | 58.1 | 35.5 | 49.3 | 67.0 | 64 | 56.6 | 23.5 | 50.7 | 62.5 | 64 | 40.4 | 26.1 | 33.9 | 46.9 |
|  |  |  |  |  |  |  |  |  |  |  |  |  |  |  |  |  |
| **[KHLPRB5] Whether CM suffers health problems: Backache, prolactin (prl) disc, sciatica** | Not mentioned | 7,262 | 88.8 | 19.1 | 88.3 | 89.2 | 7,257 | 76.2 | 17.5 | 75.8 | 76.6 | 7,278 | 70.8 | 20.7 | 70.4 | 71.3 |
|  | Mentioned | 1,502 | 73.0 | 28.0 | 71.6 | 74.5 | 1,498 | 69.3 | 20.0 | 68.3 | 70.3 | 1,503 | 56.2 | 23.9 | 55.0 | 57.4 |
|  |  |  |  |  |  |  |  |  |  |  |  |  |  |  |  |  |
| **[KHLPRB6] Whether CM suffers health problems: Cancer or leukaemia** | Not mentioned | 8,679 | 86.2 | 21.5 | 85.8 | 86.7 | 8,670 | 75.1 | 18.1 | 74.7 | 75.4 | 8,696 | 68.5 | 21.9 | 68.1 | 69.0 |
|  | Mentioned | 85 | 69.6 | 29.6 | 63.2 | 76.0 | 85 | 70.8 | 19.2 | 66.6 | 74.9 | 85 | 46.9 | 23.4 | 41.9 | 52.0 |
|  |  |  |  |  |  |  |  |  |  |  |  |  |  |  |  |  |
| **[KHLPRB7] Whether CM suffers health problems: Problems with hearing** | Not mentioned | 7,915 | 86.4 | 21.4 | 86.0 | 86.9 | 7,907 | 75.4 | 18.0 | 75.0 | 75.8 | 7,931 | 69.0 | 21.7 | 68.5 | 69.5 |
|  | Mentioned | 849 | 82.8 | 24.3 | 81.2 | 84.5 | 848 | 71.4 | 19.5 | 70.1 | 72.7 | 850 | 61.9 | 23.3 | 60.3 | 63.5 |
|  |  |  |  |  |  |  |  |  |  |  |  |  |  |  |  |  |
| **[KHLPRB8] Whether CM suffers health problems: Sight problems and glasses/lenses** | Not mentioned | 2,824 | 86.4 | 22.1 | 85.6 | 87.2 | 2,824 | 75.9 | 17.8 | 75.2 | 76.5 | 2,831 | 69.8 | 22.2 | 69.0 | 70.7 |
|  | Mentioned | 5,940 | 85.9 | 21.5 | 85.4 | 86.5 | 5,931 | 74.6 | 18.3 | 74.1 | 75.1 | 5,950 | 67.6 | 21.8 | 67.0 | 68.2 |
|  |  |  |  |  |  |  |  |  |  |  |  |  |  |  |  |  |
| **[KHLPRB9] Whether CM suffers health problems: High blood pressure** | Not mentioned | 7,433 | 87.4 | 20.2 | 87.0 | 87.9 | 7,427 | 75.7 | 17.7 | 75.3 | 76.1 | 7,445 | 70.4 | 20.9 | 69.9 | 70.9 |
|  | Mentioned | 1,331 | 78.5 | 27.3 | 77.1 | 80.0 | 1,328 | 71.2 | 20.3 | 70.1 | 72.3 | 1,336 | 56.7 | 23.9 | 55.4 | 58.0 |
|  |  |  |  |  |  |  |  |  |  |  |  |  |  |  |  |  |
| **[KHLPRB10] Whether CM suffers health problems: Migraine** | Not mentioned | 8,010 | 86.6 | 21.3 | 86.1 | 87.0 | 7,999 | 75.5 | 17.9 | 75.1 | 75.9 | 8,025 | 69.0 | 21.7 | 68.5 | 69.4 |
|  | Mentioned | 754 | 80.9 | 24.8 | 79.1 | 82.7 | 756 | 70.0 | 20.1 | 68.6 | 71.4 | 756 | 61.5 | 23.9 | 59.8 | 63.2 |
|  |  |  |  |  |  |  |  |  |  |  |  |  |  |  |  |  |
| **[KHLPRB11] Whether CM suffers health problems: Eczema/other skin problems** | Not mentioned | 8,047 | 86.6 | 21.2 | 86.1 | 87.0 | 8,039 | 75.4 | 17.9 | 75.1 | 75.8 | 8,064 | 69.0 | 21.6 | 68.6 | 69.5 |
|  | Mentioned | 717 | 80.8 | 25.5 | 78.9 | 82.6 | 716 | 70.1 | 20.3 | 68.6 | 71.6 | 717 | 60.3 | 24.7 | 58.5 | 62.2 |
|  |  |  |  |  |  |  |  |  |  |  |  |  |  |  |  |  |
| **[KHLPRB12] Whether CM suffers health problems: Chronic fatigue syn. (ME)** | Not mentioned | 8,705 | 86.4 | 21.3 | 85.9 | 86.8 | 8,696 | 75.1 | 18.0 | 74.8 | 75.5 | 8,722 | 68.6 | 21.7 | 68.2 | 69.1 |
|  | Mentioned | 59 | 44.8 | 30.0 | 37.0 | 52.6 | 59 | 57.1 | 23.2 | 51.0 | 63.1 | 59 | 23.4 | 17.9 | 18.8 | 28.1 |
|  |  |  |  |  |  |  |  |  |  |  |  |  |  |  |  |  |
| **[KHLPRB14] Whether CM suffers health problems: Stomach/bowels/gall bladder** | Not mentioned | 8,031 | 87.0 | 20.8 | 86.6 | 87.5 | 8,025 | 75.9 | 17.6 | 75.5 | 76.3 | 8,048 | 70.0 | 20.9 | 69.5 | 70.5 |
|  | Mentioned | 733 | 75.6 | 27.5 | 73.6 | 77.6 | 730 | 65.3 | 21.1 | 63.8 | 66.8 | 733 | 49.9 | 24.9 | 48.1 | 51.7 |
|  |  |  |  |  |  |  |  |  |  |  |  |  |  |  |  |  |
| **[KHLPRB15] Whether CM suffers health problems: Problems with bladder/kidneys** | Not mentioned | 8,550 | 86.6 | 21.0 | 86.2 | 87.1 | 8,541 | 75.3 | 17.9 | 74.9 | 75.7 | 8,567 | 69.0 | 21.4 | 68.5 | 69.4 |
|  | Mentioned | 214 | 64.6 | 34.3 | 59.9 | 69.2 | 214 | 62.7 | 23.3 | 59.6 | 65.8 | 214 | 42.2 | 27.0 | 38.5 | 45.8 |
|  |  |  |  |  |  |  |  |  |  |  |  |  |  |  |  |  |
| **[KHLPRB16] Whether CM suffers health problems: Cough/bringing up phlegm** | Not mentioned | 8,398 | 86.5 | 21.3 | 86.0 | 87.0 | 8,390 | 75.5 | 17.9 | 75.1 | 75.8 | 8,415 | 69.1 | 21.6 | 68.6 | 69.6 |
|  | Mentioned | 366 | 76.5 | 26.7 | 73.7 | 79.2 | 365 | 64.8 | 21.6 | 62.6 | 67.1 | 366 | 50.7 | 23.9 | 48.2 | 53.2 |
|  |  |  |  |  |  |  |  |  |  |  |  |  |  |  |  |  |
| **[MHPROBS1] Whether CM seen doc re MH last 4yrs: feeling low/depressed/sad** | Not mentioned | 7,231 | 88.3 | 19.3 | 87.8 | 88.7 | 7,228 | 78.5 | 15.3 | 78.1 | 78.8 | 7,246 | 71.3 | 20.1 | 70.8 | 71.7 |
|  | Mentioned | 1,516 | 75.8 | 28.3 | 74.4 | 77.2 | 1,510 | 58.5 | 21.3 | 57.4 | 59.6 | 1,518 | 54.5 | 25.1 | 53.2 | 55.7 |
|  |  |  |  |  |  |  |  |  |  |  |  |  |  |  |  |  |
| **[MHPROBS2] Whether CM seen doc re MH last 4yrs: feeling anxious or jittery** | Not mentioned | 8,081 | 86.9 | 20.9 | 86.4 | 87.4 | 8,075 | 76.6 | 16.8 | 76.3 | 77.0 | 8,098 | 69.7 | 21.2 | 69.2 | 70.2 |
|  | Mentioned | 666 | 76.5 | 27.3 | 74.4 | 78.6 | 663 | 55.5 | 21.9 | 53.8 | 57.1 | 666 | 51.9 | 24.9 | 50.1 | 53.8 |
|  |  |  |  |  |  |  |  |  |  |  |  |  |  |  |  |  |
| **[MHPROBS3] Whether CM seen doc re MH last 4yrs: feels anxious re objects/sits** | Not mentioned | 8,423 | 86.7 | 21.1 | 86.2 | 87.1 | 8,415 | 75.8 | 17.4 | 75.5 | 76.2 | 8,440 | 69.1 | 21.5 | 68.7 | 69.6 |
|  | Mentioned | 324 | 71.9 | 29.4 | 68.6 | 75.1 | 323 | 53.5 | 22.5 | 51.1 | 56.0 | 324 | 48.4 | 24.6 | 45.7 | 51.1 |
|  |  |  |  |  |  |  |  |  |  |  |  |  |  |  |  |  |
| **[MHPROBS4] Whether CM seen doc re MH last 4yrs: repeat certain acts/thoughts** | Not mentioned | 8,640 | 86.3 | 21.5 | 85.8 | 86.7 | 8,631 | 75.4 | 17.8 | 75.0 | 75.7 | 8,657 | 68.6 | 21.8 | 68.2 | 69.1 |
|  | Mentioned | 107 | 71.9 | 30.3 | 66.1 | 77.7 | 107 | 46.7 | 24.3 | 42.0 | 51.4 | 107 | 44.5 | 24.2 | 39.8 | 49.1 |
|  |  |  |  |  |  |  |  |  |  |  |  |  |  |  |  |  |
| **[MHPROBS5] Whether CM seen doc re MH last 4yrs: hearing or seeing things** | Not mentioned | 8,694 | 86.3 | 21.4 | 85.8 | 86.7 | 8,685 | 75.2 | 18.0 | 74.8 | 75.6 | 8,711 | 68.5 | 21.8 | 68.1 | 69.0 |
|  | Mentioned | 53 | 59.9 | 36.1 | 49.9 | 69.8 | 53 | 46.6 | 23.6 | 40.1 | 53.1 | 53 | 39.5 | 25.0 | 32.6 | 46.4 |
|  |  |  |  |  |  |  |  |  |  |  |  |  |  |  |  |  |
| **[MHPROBS6] Whether CM seen doc re MH last 4yrs: other problems affecting mood** | Not mentioned | 8,224 | 86.9 | 20.8 | 86.5 | 87.4 | 8,216 | 76.1 | 17.2 | 75.8 | 76.5 | 8,241 | 69.4 | 21.3 | 69.0 | 69.9 |
|  | Mentioned | 523 | 73.5 | 29.4 | 71.0 | 76.1 | 522 | 57.5 | 22.5 | 55.5 | 59.4 | 523 | 51.4 | 25.8 | 49.2 | 53.7 |
|  |  |  |  |  |  |  |  |  |  |  |  |  |  |  |  |  |
| **[MHPROBS7] Whether CM seen doc re MH last 4yrs: none of the above** | Not mentioned | 1,968 | 77.9 | 27.3 | 76.6 | 79.1 | 1,961 | 61.0 | 20.9 | 60.1 | 61.9 | 1,970 | 56.4 | 24.9 | 55.3 | 57.5 |
|  | Mentioned | 6,779 | 88.5 | 19.0 | 88.1 | 89.0 | 6,777 | 79.1 | 15.0 | 78.7 | 79.4 | 6,794 | 71.8 | 19.7 | 71.4 | 72.3 |
|  |  |  |  |  |  |  |  |  |  |  |  |  |  |  |  |  |
| **[MHSTILL] Whether CM still has MH problem: feeling low/depressed/sad** | Yes, most of the time, | 310 | 62.3 | 33.1 | 58.6 | 66.0 | 308 | 37.5 | 18.4 | 35.5 | 39.6 | 311 | 38.7 | 23.6 | 36.1 | 41.3 |
|  | Yes, occasionally, | 747 | 75.8 | 27.8 | 73.8 | 77.8 | 745 | 57.4 | 17.1 | 56.2 | 58.6 | 747 | 54.0 | 23.8 | 52.3 | 55.7 |
|  | No | 457 | 85.0 | 21.2 | 83.0 | 86.9 | 455 | 74.5 | 15.4 | 73.1 | 76.0 | 458 | 65.9 | 22.1 | 63.9 | 67.9 |
|  |  |  |  |  |  |  |  |  |  |  |  |  |  |  |  |  |
| **[MHSTILL2] Whether CM still has MH problem: feeling anxious or jittery** | Yes, most of the time, | 148 | 63.1 | 33.1 | 57.8 | 68.5 | 148 | 37.8 | 19.1 | 34.7 | 40.9 | 148 | 39.0 | 24.0 | 35.1 | 42.9 |
|  | Yes, occasionally, | 336 | 77.9 | 25.0 | 75.2 | 80.6 | 334 | 54.6 | 18.7 | 52.6 | 56.6 | 336 | 52.5 | 23.5 | 50.0 | 55.0 |
|  | No | 181 | 84.9 | 21.8 | 81.7 | 88.1 | 180 | 71.7 | 17.5 | 69.1 | 74.3 | 181 | 61.8 | 23.2 | 58.4 | 65.2 |
|  |  |  |  |  |  |  |  |  |  |  |  |  |  |  |  |  |
| **[MHSTILL3] Whether CM still has MH problem: feels anxious re objects/sits** | Yes, most of the time, | 79 | 61.1 | 32.7 | 53.8 | 68.4 | 79 | 39.6 | 22.1 | 34.7 | 44.6 | 79 | 36.5 | 22.7 | 31.4 | 41.6 |
|  | Yes, occasionally, | 170 | 73.1 | 28.8 | 68.7 | 77.5 | 169 | 53.6 | 19.5 | 50.6 | 56.5 | 170 | 48.8 | 24.0 | 45.1 | 52.4 |
|  | No | 75 | 80.4 | 23.5 | 75.0 | 85.8 | 75 | 68.1 | 20.2 | 63.5 | 72.8 | 75 | 60.1 | 22.2 | 55.0 | 65.2 |
|  |  |  |  |  |  |  |  |  |  |  |  |  |  |  |  |  |
| **[MHSTILL4] Whether CM still has MH problem: repeat certain acts/thoughts** | Yes, most of the time, | 43 | 60.8 | 33.2 | 50.6 | 71.0 | 43 | 36.3 | 21.3 | 29.8 | 42.9 | 43 | 36.2 | 23.5 | 28.9 | 43.4 |
|  | Yes, occasionally, | 43 | 75.8 | 27.1 | 67.5 | 84.2 | 43 | 50.8 | 24.7 | 43.2 | 58.4 | 43 | 46.8 | 22.9 | 39.8 | 53.8 |
|  | No | 21 | 86.7 | 22.0 | 76.6 | 96.7 | 21 | 59.6 | 21.5 | 49.8 | 69.4 | 21 | 56.7 | 22.6 | 46.4 | 67.0 |
|  |  |  |  |  |  |  |  |  |  |  |  |  |  |  |  |  |
| **[MHSTILL5] Whether CM still has MH problem: hearing or seeing things** | Yes, most of the time, | 6 | 37.5 | 45.5 | -10.2 | 85.2 | 6 | 25.3 | 18.7 | 5.7 | 45.0 | 6 | 20.8 | 21.8 | -2.0 | 43.7 |
|  | Yes, occasionally, | 29 | 54.9 | 35.3 | 41.5 | 68.4 | 29 | 45.3 | 21.4 | 37.2 | 53.5 | 29 | 38.2 | 22.4 | 29.7 | 46.7 |
|  | No | 18 | 75.3 | 29.3 | 60.7 | 89.8 | 18 | 55.8 | 24.5 | 43.6 | 68.0 | 18 | 47.8 | 27.5 | 34.1 | 61.5 |
|  |  |  |  |  |  |  |  |  |  |  |  |  |  |  |  |  |
| **[MHSTILL6] Whether CM still has MH problem: other problems affecting mood** | Yes, most of the time, | 144 | 62.7 | 33.1 | 57.3 | 68.2 | 143 | 43.3 | 22.3 | 39.7 | 47.0 | 144 | 39.6 | 25.0 | 35.4 | 43.7 |
|  | Yes, occasionally, | 254 | 73.9 | 28.5 | 70.4 | 77.4 | 254 | 57.2 | 19.3 | 54.8 | 59.6 | 254 | 50.7 | 24.4 | 47.7 | 53.7 |
|  | No | 123 | 85.0 | 21.1 | 81.3 | 88.8 | 123 | 74.2 | 16.8 | 71.2 | 77.2 | 123 | 66.5 | 21.6 | 62.7 | 70.4 |
|  |  |  |  |  |  |  |  |  |  |  |  |  |  |  |  |  |
| **[MHSPEC] Whether CM seen doctor about mental health problem in last year** | Yes | 1,138 | 74.1 | 29.0 | 72.4 | 75.8 | 1,133 | 56.2 | 21.2 | 54.9 | 57.4 | 1,139 | 51.3 | 25.0 | 49.9 | 52.8 |
|  | No | 830 | 83.0 | 23.9 | 81.4 | 84.7 | 828 | 67.7 | 18.6 | 66.4 | 68.9 | 831 | 63.3 | 23.1 | 61.7 | 64.9 |
|  |  |  |  |  |  |  |  |  |  |  |  |  |  |  |  |  |
| **[KHLDSBL] Whether CM registered disabled (See DV ND8KHLDS)** | Yes | 353 | 38.9 | 33.5 | 35.4 | 42.4 | 349 | 59.0 | 22.7 | 56.6 | 61.4 | 353 | 33.4 | 25.1 | 30.8 | 36.0 |
|  | No | 7,471 | 87.4 | 18.9 | 86.9 | 87.8 | 7,468 | 74.9 | 17.8 | 74.5 | 75.3 | 7,486 | 68.4 | 20.8 | 67.9 | 68.8 |
|  |  |  |  |  |  |  |  |  |  |  |  |  |  |  |  |  |
| **[KHLLT] Whether health limits everyday activities (See DV ND8KHLLT)** | Yes | 1,339 | 56.7 | 29.6 | 55.1 | 58.3 | 1,332 | 61.6 | 21.5 | 60.5 | 62.8 | 1,340 | 38.9 | 22.0 | 37.7 | 40.1 |
|  | No | 6,483 | 91.0 | 14.6 | 90.7 | 91.4 | 6,483 | 76.8 | 16.5 | 76.4 | 77.2 | 6,497 | 72.5 | 17.4 | 72.1 | 72.9 |
|  |  |  |  |  |  |  |  |  |  |  |  |  |  |  |  |  |
| **[KHL2HOP] Number of times visited hospital as out-patient since last interview** | None | 3,768 | 90.7 | 17.0 | 90.2 | 91.3 | 3,764 | 77.9 | 16.6 | 77.4 | 78.4 | 3,775 | 74.9 | 17.5 | 74.3 | 75.4 |
|  | One or two | 2,328 | 89.1 | 17.2 | 88.4 | 89.8 | 2,327 | 75.7 | 16.9 | 75.0 | 76.4 | 2,332 | 71.5 | 19.0 | 70.7 | 72.2 |
|  | Three to five | 1,087 | 84.3 | 21.6 | 83.0 | 85.6 | 1,083 | 73.7 | 18.7 | 72.6 | 74.8 | 1,087 | 64.8 | 21.7 | 63.5 | 66.1 |
|  | Six to ten | 737 | 77.8 | 26.6 | 75.8 | 79.7 | 736 | 70.8 | 19.8 | 69.3 | 72.2 | 739 | 56.5 | 23.6 | 54.8 | 58.2 |
|  | More than ten | 808 | 66.0 | 31.3 | 63.8 | 68.2 | 809 | 65.5 | 21.5 | 64.0 | 67.0 | 812 | 44.7 | 26.1 | 42.9 | 46.5 |
|  | Don't know | 37 | 78.5 | 28.0 | 69.2 | 87.8 | 37 | 70.3 | 22.8 | 62.8 | 77.9 | 37 | 61.0 | 25.3 | 52.6 | 69.4 |
|  |  |  |  |  |  |  |  |  |  |  |  |  |  |  |  |  |
| **[KHOSP] Whether been in hospital/clinic as day/in-patient since last interview** | Yes | 2,451 | 78.3 | 27.5 | 77.2 | 79.4 | 2,451 | 70.9 | 20.1 | 70.1 | 71.6 | 2,458 | 59.5 | 25.3 | 58.5 | 60.5 |
|  | No | 6,313 | 89.1 | 18.1 | 88.7 | 89.5 | 6,304 | 76.6 | 17.0 | 76.2 | 77.0 | 6,323 | 71.8 | 19.5 | 71.3 | 72.2 |

**Table S2: Results of multiply imputed and weighted regression modelling of SF-36 outcomes for each social class theory.**

| **Social class theory** |  | **Physical functioning** | | **Emotional well-being** | | **General health** | |
| --- | --- | --- | --- | --- | --- | --- | --- |
| **(*1). Social background and early life circumstances:*** | **DF** | **F** | **p-value** | **F** | **p-value** | **F** | **p-value** |
| Gender | 1 | 52.3 | <0.001 | 59.3 | <0.001 | 5.2 | 0.022 |
| Father's social class | 5 | 6.3 | <0.001 | 2.0 | 0.102 | 5.9 | <0.001 |
| Grandparent's social class | 5 | 0.4 | 0.641 | 1.1 | 0.348 | 1.3 | 0.270 |
| Mother's education | 3 | 3.4 | 0.020 | 3.0 | 0.031 | 4.5 | 0.004 |
| Father's education | 3 | 4.0 | 0.010 | 0.6 | 0.591 | 2.5 | 0.064 |
| Access to free school meals | 1 | 17.7 | <0.001 | 7.7 | 0.006 | 11.1 | 0.001 |
| Family financial hardships | 2 | 8.2 | <0.001 | 11.8 | <0.001 | 13.4 | <0.001 |
| Over-crowdedness in childhood | 3 | 4.2 | 0.006 | 1.2 | 0.292 | 0.4 | 0.711 |
| Access to household amenities | 3 | 1.1 | 0.337 | 1.3 | 0.287 | 2.9 | 0.034 |
| ***(2). Habitus and distinction:*** |  |  |  |  |  |  |  |
| Gender | 1 | 73.5 | <0.001 | 55.9 | <0.001 | 0.4 | 0.392 |
| Cognitive Ability (Draw-A-Man Test score) | 1 | 50.0 | <0.001 | 17.6 | <0.001 | 18.6 | <0.001 |
| Number of leisure activities in childhood | 5 | 1.0 | 0.336 | 2.7 | 0.046 | 1.2 | 0.289 |
| Imagined occupation at 25 | 7 | 2.0 | 0.063 | 4.2 | <0.001 | 1.4 | 0.239 |
| Aspirations/plans after leaving secondary school | 3 | 4.1 | 0.011 | 1.6 | 0.210 | 2.8 | 0.062 |
| Teachers’ view: Child poor speech rating | 3 | 5.6 | 0.001 | 3.7 | 0.015 | 2.2 | 0.103 |
| Teachers Ability ratings-Maths & English | 1 | 36.9 | 0.000 | 0.0 | 0.320 | 15.0 | <0.001 |
| TV watching | 5 | 2.9 | 0.034 | 2.8 | 0.022 | 5.1 | <0.001 |
| Book Readership | 5 | 6.1 | <0.001 | 0.2 | 0.735 | 1.1 | 0.369 |
| Newspaper readership | 3 | 2.3 | 0.103 | 1.4 | 0.248 | 2.3 | 0.086 |
| Voted in general election | 4 | 1.6 | 0.193 | 1.9 | 0.126 | 0.9 | 0.371 |
| Voting intentions | 6 | 4.5 | <0.001 | 3.5 | 0.004 | 3.8 | 0.002 |
| Trades union membership | 1 | 0.0 | 0.204 | 0.0 | 0.294 | 0.1 | 0.151 |
| Attendance at religious meetings | 4 | 1.1 | 0.321 | 0.9 | 0.401 | 1.3 | 0.264 |
| ***(3). Exploitation and domination:*** |  |  | |  | |  | |
| Gender | 1 | 59.5 | <0.001 | 71.2 | <0.001 | 3.0 | 0.084 |
| Capital Accrued (1-least to 5-most) | 4 | 1.5 | 0.223 | 2.1 | 0.091 | 2.4 | 0.067 |
| Main finance to buy home | 3 | 0.5 | 0.515 | 0.5 | 0.228 | 1.2 | 0.283 |
| Main source of property purchase price | 5 | 2.6 | 0.089 | 0.8 | 0.435 | 1.7 | 0.192 |
| No. rooms (apart from the bathroom & kitchen) | 6 | 3.4 | 0.005 | 7.3 | <0.001 | 4.1 | 0.001 |
| Mortgage as percent of house price | 6 | 1.7 | 0.178 | 0.9 | 0.362 | 2.3 | 0.044 |
| Tenure | 6 | 49.9 | <0.001 | 39.8 | <0.001 | 53.9 | <0.001 |
| ***(4). Location within market relations:*** |  |  | |  | |  | |
| Gender | 1 | 29.4 | <0.001 | 28.7 | <0.001 | 14.7 | <0.001 |
| Own Social Class | 5 | 14.4 | <0.001 | 13.3 | <0.001 | 12.6 | <0.001 |
| Type of Secondary School | 4 | 2.1 | 0.095 | 0.5 | 0.610 | 2.0 | 0.102 |
| Highest Qualifications | 5 | 16.2 | <0.001 | 7.3 | <0.001 | 7.9 | <0.001 |
| No. of times on benefits in adulthood | 4 | 73.0 | <0.001 | 42.5 | <0.001 | 63.5 | <0.001 |
| No. of unemployment episodes in adulthood | 3 | 3.7 | 0.013 | 4.5 | 0.004 | 10.3 | <0.001 |
| Age left full-time continuous education | 1 | 13.9 | <0.001 | 0.0 | 0.214 | 6.8 | 0.012 |
| Family income per week (net) | 1 | 4.0 | 0.066 | 17.9 | <0.001 | 10.7 | 0.002 |
| Savings and Investments (£1000's) | 1 | 0.0 | 0.235 | 3.7 | 0.056 | 0.3 | 0.424 |
| Debt (£1000's) | 1 | 1.9 | 0.162 | 0.0 | 0.200 | 0.9 | 0.193 |

Level of significance: *p*<0.05; *Num, DF=numerator degrees of freedom.*
